# Stability and change in latent movement behaviour profiles during adolescence and links with future depressive symptoms

**DOI:** 10.1038/s41598-025-04466-7

**Published:** 2025-07-01

**Authors:** Christopher Knowles, Gavin Breslin, Angela Carlin, Kyle Paradis, Stephen Shannon

**Affiliations:** 1 Manchester Institute of Education, University of Manchester, Manchester, M15 6JA UK; 2 School of Psychology, Queen’s University Belfast, Belfast, BT9 1NN UK; 3 Sport and Exercise Sciences Research Institute, School of Sport and Exercise Science, Ulster University, Belfast, BT15 1ED UK; 4 Bamford Centre for Mental Health and Well-being, School of Psychology, Ulster University, Belfast, BT48 7JL UK

**Keywords:** ALSPAC, Adolescence, Depressive symptoms, Latent transition analysis, Light physical activity, Mixture modelling, MVPA, Sedentary behaviour, Human behaviour, Risk factors, Depression, Public health, Epidemiology

## Abstract

Movement behaviours are known to influence long-term health and wellbeing. During adolescence, daily movement diminishes while depressive symptoms increase. The principle of time displacement dictates that when awake, altering time spent in one movement state (i.e., Sedentary, Light Physical Activity or Moderate-to-Vigorous Physical Activity) necessarily alters time spent in others, yet few studies examine daily movement behaviour using composite behavioural profiles. The current study used Random Intercepts Latent Transition Analysis (RI-LTA) to: identify latent profiles of movement behaviour age 12, 14 and 16; examine transition probabilities and predictors; and assess whether transition patterns contributed to depressive symptoms age 18 and 22. Data were drawn from 4,964 participants in the Avon Longitudinal Study of Parents and their Children. Movement behaviours were assessed via Actigraph (AM7164-2.2) accelerometery; depressive symptoms via the Short Mood and Feelings Questionnaire. A 3 × 3 non-invariant RI-LTA model fit the data well (BIC = 410417; Entropy = 0.902) distinguishing *Maximal-*,* Moderate-*, and *Minimal-Movers*. Once accounting for non-invariance (i.e., that all profiles moved less over time), the *Moderate-Mover* profile presented as highly stable across adolescence. Females, and those with higher BMI and more educated parents were likely to transition to profiles characterised by lesser movement. Transition patterns containing a period of minimal movement predicted worse depressive symptoms at ages 18 and 22. Notably, maximal movement age 12 conferred protection against depressive symptoms age 22, even when followed by later declines. Findings highlight the value of maximising movement early, underscoring the importance of early intervention strategies to promote movement and reduce young peoples’ depressive symptoms long-term.

**Supplementary Information:**

The online version contains supplementary material available at 10.1038/s41598-025-04466-7.

## Introduction

Of the estimated 970 million people currently living with a mental health condition globally, around 280 million (28.9%) are living with a depressive disorder^1^. As a percentage of the total global population, this equates to roughly 3.8%, a prevalent rate that has endured for decades^1^. While a major point of concern, the suffering caused by depression likely extends much further. Stigma and discrimination disincentivise many with a disorder from seeking help, meaning the true prevalence is likely far greater. Moreover, an untold number of people also experience sub-clinical symptoms that go unrecognised and untreated and can manifest in clinically relevant symptomology later in life. Given limitations in case detection and diagnostic thresholds, research examining the full continuum of depressive symptomatology – including sub-clinical presentations, which are a major contributor to disability across the lifespan – is essential for capturing the true scope of population mental health^1,2^.

Adolescence is closely linked to the emergence of depressive symptoms. Reports show that the peak age of onset of depressive symptoms is 15.5 years of age^2^ and that early intervention can be pivotal in preventing symptom escalation^3,4^. This life stage is characterized by heightened neurological and developmental plasticity making young people receptive to behaviour change and positive habit formation^5,6^. Patterns of health and risk behaviour established in these formative years can shape long-term health trajectories and impact the likelihood a young person experiences depressive symptoms and/or reports favourable wellbeing^7–10^. Physical Activity (PA) and sedentary behaviour are particularly salient health/risk behaviours due to high prevalence of inactivity and sedentary behaviour in this age group^11^.

While regular Moderate-to-Vigorous Physical Activity (MVPA) has long been recognised as a low-risk, low-cost, method of managing depressive symptoms (and indeed, all-round health and wellbeing)^12^, associations with sedentary behaviour are more nuanced. Adolescent sedentary behaviour has become increasingly synonymous with screen time and social media use which itself has been linked to suboptimal health including a “*Wellness Weary*” behavioural pattern comprising little PA, insufficient sleep, and low-quality diet^10^. Contrastingly, evidence from the Health Behaviours in School-Aged Children study indicates that heavy but not problematic social media use can foster feelings of peer support and social connectedness for some individuals, but can exacerbate feelings of loneliness and provide a context for bullying victimisation for others^13,14^. Inconsistent effects of screen time and social media use are also reported in other studies some of which have linked high social media exposure to elevated risk of depressive symptoms and suicidality^15^. Meanwhile, a network analysis that integrated social media within a dynamic system of inter-related components (mental health, family dynamics, peer support and the school environment) placed social media use among the least influential contributors to adolescent mental health^16^. Heterogeneity of effects is also reflected across other sedentary behaviours including reading for enjoyment which generally presents as beneficial for adolescent mental health^17^, while prolonged TV viewing has been linked to elevated depressive symptoms long-term^18^. While international guidelines recommend minimising sedentary time for optimal physical health^19^; evidently, the same universal recommendation cannot be directly applied to mental health outcomes.

Complexity in the association between physical movement and depressive symptoms in adolescence is compounded by a critical lack of insight into the effects of Light Physical Activity (LPA)^20^. A growing body of evidence demonstrates that reductions in depressive symptoms can be achieved via LPA in adulthood^21^ however, care should be taken not to generalise these findings to younger populations. Young people experience many psychological, social, and developmental challenges distinct from those faced in adulthood including academic pressure and ongoing identity formation, influencing their motivation and ability to be active. Similarly, a fit and healthy teenager may find LPA insufficiently taxing to induce a sense of task-mastery; and therefore less wellbeing supportive^22^. Nevertheless, there is some evidence that LPA has mental health benefits for youth^23^ and given that less than half of young people in England currently meet MVPA guidelines^11^, efforts to get the nation moving will likely continue to incorporate LPA as part of the solution. There is, therefore, an ongoing need to better understand associations with all types, intensities, and volumes of movement behaviour in adolescence.

PA and sedentary behaviour are not simply two sides of the same coin and are rightly treated as independent variables in respective fields of research. However, the necessity that when awake, one *must* be either sedentary, lightly-, or moderate-to-vigorously active means PA and sedentary behaviour are inextricably linked^24^. The finitude of time dictates that when awake, one *must* decrease time spent in at least one state in order to boost time spent in another. Accordingly, recommendations for epidemiologists now emphasise that analytical approaches integrate all forms of movement into composite patterns of daily behaviour^25,26^. Such approaches align with Health Lifestyle Theory and the Overflow Hypothesis both of which speak to the effect of co-dependence among concomitant health-influencing behaviours^27,28^.

Failure to holistically evaluate the composition of adolescent movement behaviours and the impact of transitions between movement profiles risks mischaracterising the potential of movement-based public health strategies, and advocacy for preventative action ends up less effective than anticipated. For example, one study performing compositional data analysis presents evidence that displacing sedentary time with MVPA yields more pronounced physical health benefits than if displacing LPA, which itself offers some advantages for disease markers (e.g., cardiovascular capacity, obesity)^24^. Although associations with mental health are less clear cut due to the potential for sedentary activities to be both supportive (e.g., reading for enjoyment) *and* detrimental to wellbeing (e.g., problematic social media use)^13^, emerging person-centred studies suggest the benefits of adolescent movement for depressive symptoms are dependent on a *synergistic interaction* and the ideal combination of co-dependent movement behaviours performed throughout the day^29^. Examining MVPA, LPA, and sedentary behaviour as component parts of an integrated movement profile therefore provides insight into how different combinations of these behaviours impact both physical and mental health, thereby aiding development of more nuanced, targeted, and effective public health strategies.

Person-centred approaches assume population heterogeneity and profiles individuals appropriately according to shared characteristics, yielding advanced theoretical knowledge applicable to public health advocates where variable-centred studies cannot^30,31^. In particular, Latent Transition Analysis (LTA) probabilistically assigns individuals to the profile with which they most closely correspond repeatedly across multiple timepoints, and calls on relevant predictor variables to estimate the likelihood of transitioning profiles for subgroups of the population.

Evidence suggests “high-decreasers” (those most physically active in childhood but decline by late adolescence) experience fewer depressive symptoms in early adulthood than those who only started moving more later in adolescence^9^. This infers a portion of the variance in depressive symptoms among individuals exhibiting similar volumes of movement may be explained by a lagged and pervasive effect of historic movement-based behaviours. A powerful feature of LTA in this regard, is it enables examination of how *transition patterns* can account for variance in mental health outcomes by considering historic volumes of movement and the path one took to arrive at a particular latent profile^32^. LTA provides an insightful framework to better understand why comparable activity levels at a single timepoint do not consistently predict mental health in adolescence.

Recent methodological advancements now suggest including random intercepts within LTA models can greatly enhance model accuracy by parsing stable, individual traits from other factors influencing profile changes^33^. The present study is among the first to utilise Random Intercepts Latent Transition Analysis (RI-LTA) generally, and the very first to use accelerometer data to objectively assess holistic movement profiles within a RI-LTA framework. By tracking changes in movement profiles across three stages of adolescence (age 12, 14, 16) this study offers unique insight into how transitions in movement behaviour influence depressive symptoms in late adolescence (age 18) and early adulthood (age 22).

The purpose of the current study was to establish: (i) distinct movement behaviour profiles at multiple timepoints across adolescence; (ii) predictors of profile transitions; and (iii) the extent to which transition patterns predict future depressive symptoms. Depressive symptoms at age 18 and 22 were evaluated to determine the extent to which movement behaviours may influence depressive symptoms over multiple timeframes. Importantly, the current study does not integrate time spent asleep within movement profiles due to the unavailability of device-based sleep data, meaning the focus is on *waking movement behaviour* rather than complete 24-hour behavioural surveillance. However, adjustments for wear time are made to account for variability in sleep patterns (among other reasons for device non-wear), the limitations of which are discussed below. Sex, body mass index (BMI), and parental education are included as covariates to account for known sociodemographic and health-related differences in movement behaviours and depressive symptoms during adolescence. Sex differences have been consistently observed in PA patterns and the prevalence of depressive symptoms (e.g., boys typically engage in more MVPA, while girls report higher rates of depressive symptoms)^34^. BMI may influence movement behaviours due to physical and/or psychosocial barriers to activity, and is independently associated with mental health outcomes^35^. Parental education serves as a proxy for socioeconomic status, which is a robust determinant of both adolescent activity levels and risk for depressive symptoms^36^.

**Fig. 1:**
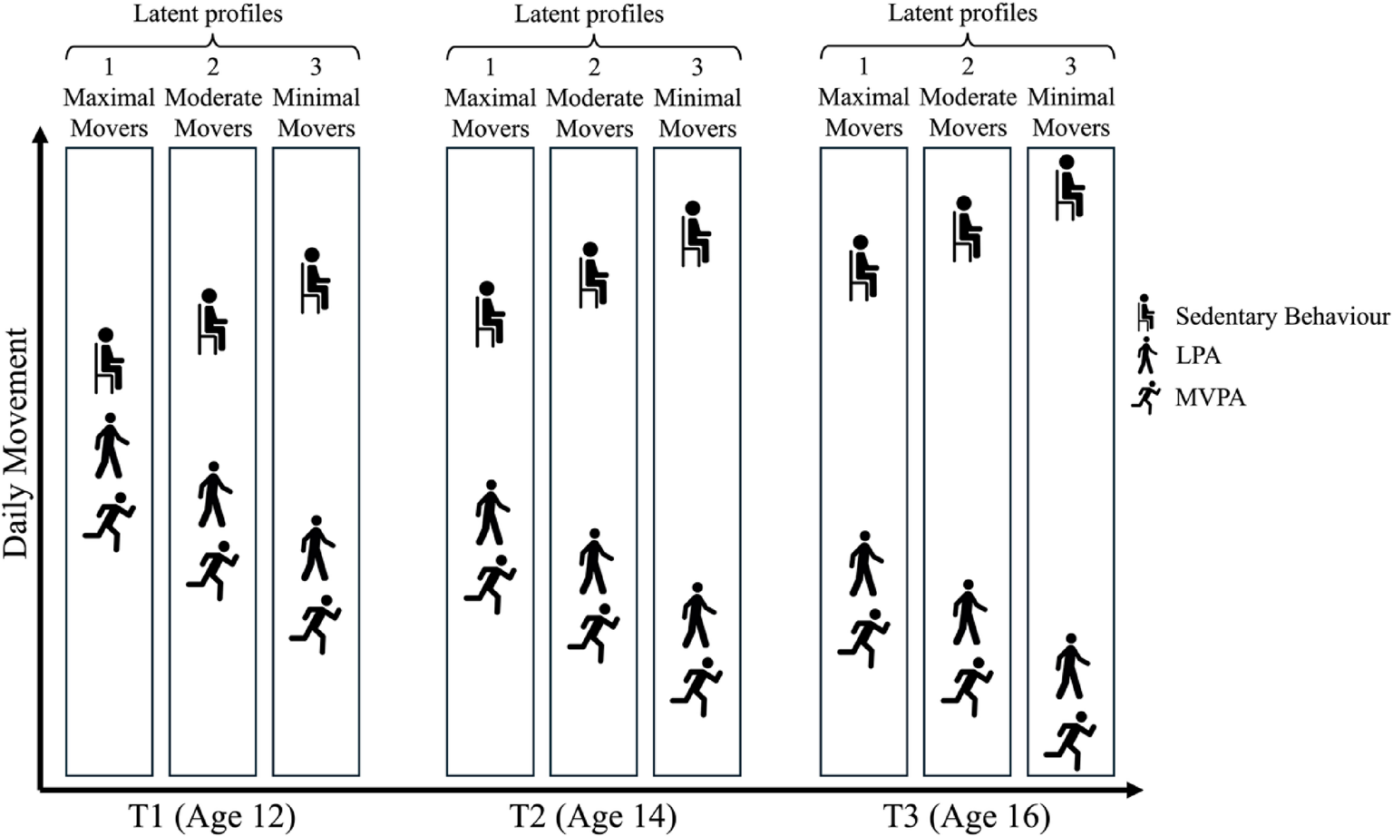
Predicted profile characteristics relating to H^1^ and H^2^. Each bar represents an hypothesised movement profile and are clustered by age at measurement. Each profile is associated with less movement than its equivalent at the timepoint prior.

**Fig. 2:**
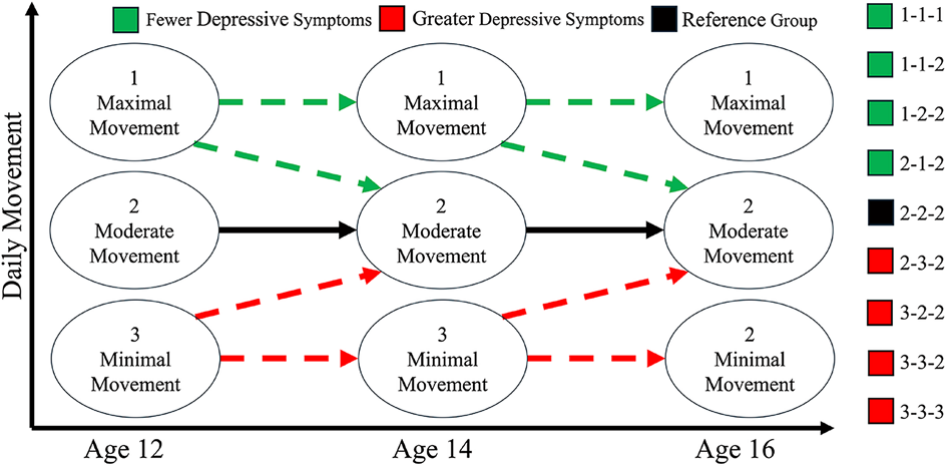
Hypothesised differences in depressive symptoms among those exhibiting different transition patterns proposed for H^4^ & H^5^.

Health Lifestyle Theory posits that barriers to engagement in one health behaviour likely to extend to other health behaviours^28^. We therefore did not expect to find profiles showing high levels of MVPA paired with low levels of LPA (or vice versa). Instead, we anticipated profiles where both MVPA and LPA would increase in tandem, and antagonistically to sedentary behaviour [H^1^]. In line with extant literature, we hypothesised the sample would become more sedentary and less active over time [H^2^]. We hypothesised that female sex [H^3a^], higher BMI [H^3b^], lower parental education [H^3c^] and higher baseline depressive symptoms [H^3d^] would significantly increase the likelihood of transitioning to profiles characterised by lesser movement. We hypothesised that compared to those exhibiting moderate volumes of movement consistently over time, those with a history of greater movement would report fewer depressive symptoms at age 18 [H^4a^] and age 22 [H^4b^], and that those with a history of lesser movement would report greater depressive symptoms at age 18 [H^5a^] and age 22 [H^5b^]. See Figs. 1 and 2 for illustrative examples of hypothesised profile characteristics and differences in depressive symptoms. While we did not hypothesise identifying a specific number of profiles at any timepoint, in both figures, three profiles are depicted to support interpretation.

## Methods

### Participants

Participants were part of the Avon Longitudinal Study of Parents and Children (ALSPAC)^37–39^. All pregnant women resident in Avon, UK with expected delivery dates between 1st April 1991 and 31st December 1992 were invited to take part. After subsequent attempts to bolster the initially enrolled sample of 15,454 pregnant women, the total number of foetuses alive at one year of age was 14,901.

Study data were collected and managed using Research Electronic Data Capture (REDCap) tools hosted at the University of Bristol^40^. REDCap is a secure, web-based software platform designed to support data capture for research studies. Further information regarding research ethics can be found via the study website^41^. The ALSPAC study website contains details of all available data through a fully searchable data dictionary and variable search tool^42^. The analytical sample comprised all participants with at least 4 valid days (≥ 600 min) of accelerometer data containing ≥ 3 weekdays and ≥ 1 weekend day at ≥ 1 timepoint were included in analysis (*n* = 4,964).

### Physical activity

PA was measured using Actigraph AM7164 2.2 accelerometers [Actigraph LLC, Fort Walton Beach, FL, USA] in 2005–2006 when participants were 11.7 years of age (henceforth referred to as age 12; T1) and again when age 13.9 (henceforth 14 years; T2), and age 15.5 (henceforth 16 years; T3). On each occasion, participants were instructed to wear the device during waking hours for up to 7 consecutive days except when doing aquatic activities (e.g., bathing, showering, water sports). The time of day devices were worn and taken off and the length of time (minutes) spent swimming or cycling was recorded via self-reported timesheets. Time spent sedentary, in LPA, and MVPA was quantified using count per minute (cpm) thresholds determined in a previous calibration study (sedentary behaviour < 200 cpm; LPA ≥ 200cpm, < 3600 cpm; MVPA ≥ 3600 cpm)^43^. Raw accelerometer counts were recorded in 60-second epochs. ALSPAC accelerometer data had a pre-imposed non-wear cut-point of any period > 10 min with consecutive zero counts^43,44^.

### Depressive symptoms

Data on depressive symptoms were collected on several occasions throughout adolescence and early adulthood. The current study uses data collected during late adolescence (age 18) and early adulthood (age 22) to give an indication of the longevity of effects and perseverance of associations over time. Symptoms were quantified via the Short Mood and Feelings Questionnaire (SMFQ)^45^. The SMFQ is a 13-item self-report measure of depressive symptoms over the past two weeks with responses scored on a 3-point scale: 0 = Not True; 1 = Sometimes True; and 2 = True. Total scores range from 0 to 26 with higher scores indicating greater symptom severity. The SMFQ has been validated for use as a screening tool for depression in adolescence^46^ and early adulthood^47^ with high construct, content, and criterion validity. The SMFQ had high internal reliability at each timepoint in the present sample (α = 0.84 to 0.90).

### Covariates

Data on sex (male/female), BMI and parental education were collected via questionnaires age 12. Participants also reported their depressive symptoms via the SMFQ around age 13. On average, participants first reported their depressive symptoms 56 weeks after first wearing accelerometers. Crucially, symptoms were reported before the second accelerometry timepoint and well before the typical peak age of onset (i.e., 15.5 years), such that few participants would be expected to exhibit high symptom severity at this age^2^. As a result, controlling for symptoms at this stage helps to limit the likelihood that outliers with unusually high symptoms early in life exert undue influence on observed associations.

### Data handling

Missing data were handled using Multiple Imputation by Chained Equations (MICE) in the Statistical Package for the Social Sciences version 28 [IBM Corp., Amonk, NY, USA]. Twenty imputed datasets were generated with aggregated scores derived following the Bar Procedure^48^. SMFQ scores were positively skewed hence, missing values were imputed using Predictive Mean Matching^49^. A complete case sensitivity analysis was also conducted to establish the extent to which attrition and data imputation impacted the structure of profiles and sample distribution (supplementary material).

### Statistical analysis

Analyses were performed using Mplus version 8.9 in four key stages^50^.

### Stage one: profile enumeration at each timepoint

A sequence of cross-sectional Latent Profile Analyses were performed age 12 (T1), 14 (T2), and 16 (T3). Models were fit using only latent profile indicators without adjusting for covariates^51^. Solutions with *k + 1* profiles were enumerated up to a 10-profile solution. Relative fit indices were used to identify the best fitting model: *Akaike Information Criterion* (AIC); *Bayesian Information Criterion* (BIC); *Sample Size Adjusted BIC* (ssaBIC); and the *Lo–Mendell–Rubin Adjusted Likelihood Ratio Test* (LMRa). For AIC, BIC, and ssaBIC, lower values represent better fit. An elbow plot was generated to identify the point at which increased model complexity yielded diminished returns in model fit^51^. Significant LMRa tests indicate the *k* solution fits *better* than that containing *k*–1 profiles^52^. The proportional distribution of the sample was also evaluated to qualify stability, interpretability and generalisability of each model. Solutions with the smallest profiles were regarded as least stable. Although not a fit index, classification entropy is reported with values > 0.80 considered clear separation between profiles^51^. The best fitting LPAs were advanced and combined into a single LTA model with profiles at each timepoint estimated simultaneously.

### Stage two: model modifications, measurement invariance and inclusion of random intercepts

Fit statistics were compared between the regular LTA and RI-LTA models (both invariant and non-invariant) to quantitatively validate that the more complex RI-LTA models were a better fit^53^. Only BIC was used to assess fit of RI-LTA models, in line with current recommendations^33^. Satorra-Bentler Scaled Mean-adjusted Chi Square tests of model fit formally tested the assumption of measurement invariance for regular LTA and RI-LTA models^54^. Satorra-Bentler tests determine whether a more complex non-invariant (comparison) model, within which measurement parameters (i.e., profile indicators) are freely estimated, is a significantly better fit than a simpler invariant (nested) model with parameters fixed to their T1 values at all timepoints. Non-significant tests suggest the simpler invariant model is preferable. All stage two models were adjusted for accelerometer wear time at each timepoint to reduce risk of bias in profile estimation and account for differences in sleep (the major contributor to device non-wear). As a further sensitivity analysis, this stage was also conducted without adjusting for wear time to illustrate the extent to which failure to make such adjustments may bias parameter estimates for future studies (supplementary material).

### Stage three: profile transition probabilities and their predictors

Transition probabilities were obtained by regressing profile membership at T2 on membership at T1, then T3 membership on T2. All parameters were then fixed to their final starting values such that the structure of profiles remained unchanged when introducing auxiliary predictors to the model^50,51^. Predictors of profile transitions were fit simultaneously to account for potential between-predictor interactions. As risk of Type II Error increases when adding multiple predictors simultaneously, sensitivity analyses were performed with predictors tested individually in separate models (supplementary material).

### Stage four: comparing depressive symptoms across transition probabilities

Where possible, differences in depressive symptoms at ages 18 and 22 were assessed via Wald tests to compare estimated SMFQ scores for each transition pattern (e.g., 2→2→2 versus 3→3→2) for minimally and fully adjusted models (refer to Fig. 2 for support interpreting transition patterns). The largest stable transition pattern was used as reference against which all viable unstable transition patterns (i.e., increasing/decreasing/fluctuating) were compared. Some transition patterns are not reported as they contained very few participants (e.g., 3→1→2) and were considered unreliable.

## Results

### Estimated sample means and descriptive statistics

The analytical sample comprised 4,964 participants (52.48% female) with an average BMI at age 12 of 19.73 (*sd =* 3.24). Mean accelerometer wear time decreased across waves: 77.99 (16.18) hours at age 12, 65.36 (22.56) hours at age 14, and 55.06 (21.09) hours at age 16. Volumes of movement behaviour observed in the analytical sample are consistent with those reported in the Mattocks et al. study^44^, which informed key decisions in the ALSPAC protocol. At age 12, participants accumulated an average of 23.69 (14.19) mins/day of MVPA, 327.97 (53.56) mins/day of LPA, and 428.85 (60.69) mins/day of sedentary behaviour. At age 14, average values were 25.87 (12.91) mins/day MVPA, 280.35 (41.35) mins/day LPA, and 486.83 (49.64) mins/day sedentary. At age 16, participants recorded 26.74 (10.18) mins/day MVPA, 246.81 (29.09) mins/day LPA, and 524.16 (33.94) mins/day sedentary. The proportion of ‘Active’ participants (i.e., those meeting the UK Chief Medical Officers’ recommendation of ≥ 60 min/day MVPA) was low at all ages: 2.22% age 12, 2.24% age 14, and 1.09% age 16. Comparatively, 25.79% (age 12), 27.28% (age 14) and 26.09% (age 16) were ‘Fairly Active’ (defined as 30–59 min MVPA)^55^, and 72.00% (age 12), 70.49% (age 14) and 72.82% (age 16) were ‘Inactive’ (defined as < 30 min MVPA)^55^. Average SMFQ scores rose from 3.63 (3.65) at age 13 to 5.23 (4.38) at age 18 followed by a decline to 3.61 (4.23) at age 22.

### Stage one: profile enumeration

**Fig. 3:**
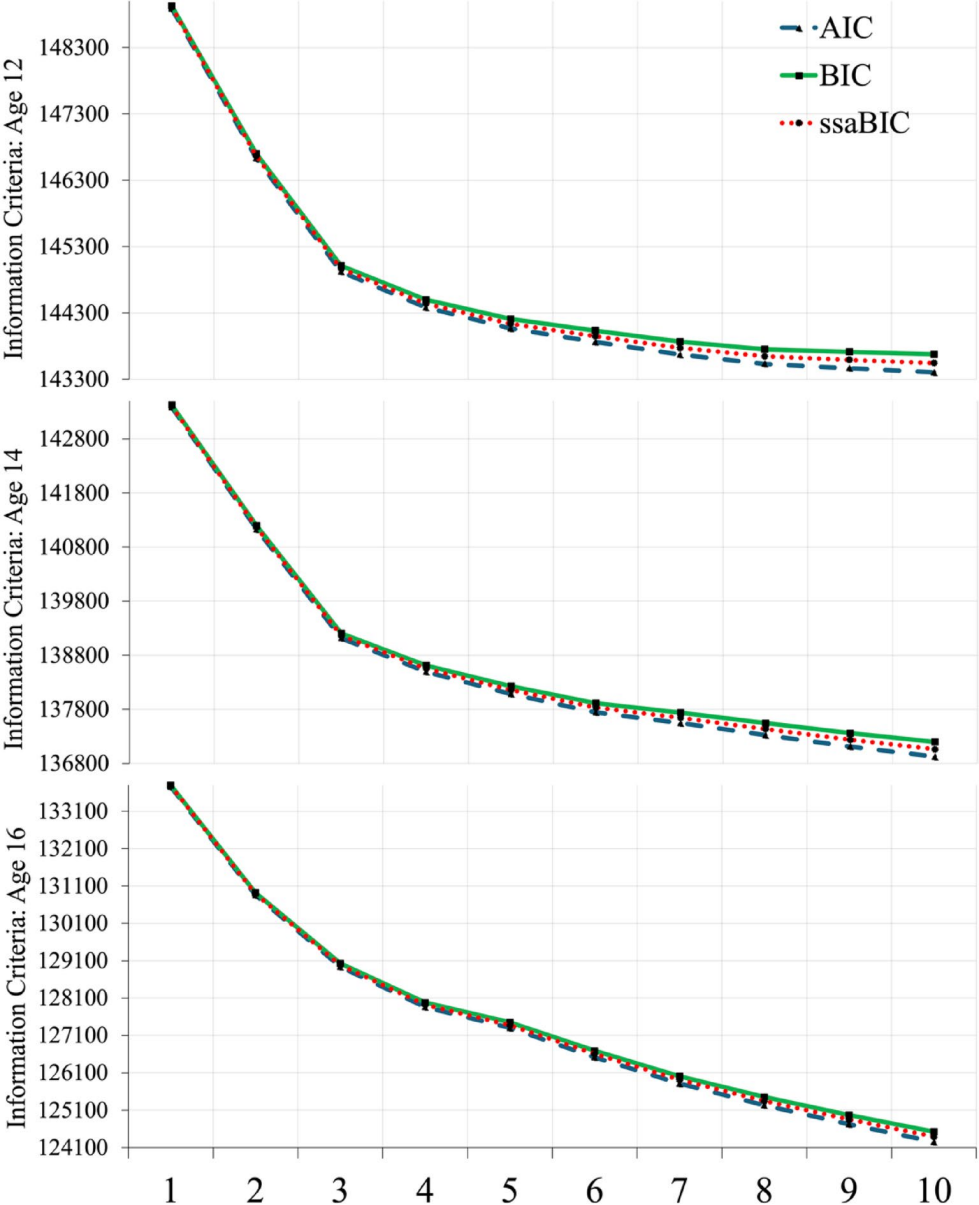
Elbow plots illustrating model fit cross-sectional latent profile models with k + 1 solutions.

Fit statistics for each timepoint are provided in Table 1. A clear elbow was visible at T1 for three profiles inferring beyond this point, increased model complexity yielded diminished returns in fit (Fig. 3). The three-profile solution was well distributed with three large groups identified, unlike the four-profile solution which contained a group comprising only 5% of the sample. At T2, an elbow was observed at three profiles (Fig. 3) and again, the sample was well distributed. Unlike T1 and T2, no clear elbow was observed at T3 (Fig. 3). However, a series of LMRa likelihood ratio tests indicated three profiles were better than two, but four profiles were not better than three. Likewise, the four-profile solution contained an unstable group comprising just 1% of the sample. For these reasons, three-profile solutions were selected as those which fit the data best at T1, T2 and T3 independently.

**Table 1:** Model fit indices for cross-sectional latent profiles of adolescent movement behaviour.

Classes	LL	AIC	BIC	ssaBIC	LMRa	Entropy	Class proportions based on estimated posterior probabilities (%)
Age 12							
1	−74,439	148,890	148,929	148,910	-	-	100
2	−73,306	146,632	146,697	146,665	0.000	0.745	76, 24
3	−72,446	144,920	145,011	144,967	0.000	0.804	63, 22, 15
4	−72,172	144,381	144,498	144,441	0.000	0.826	61, 22, 12, 5
5	−72,010	144,065	144,208	144,138	0.001	0.835	59, 20, 14, 5, 2
6	−71,905	143,863	144,033	143,950	0.000	0.847	58, 21, 13, 5, 2, 1
7	−71,805	143,671	143,866	143,771	0.000	0.828	53, 21, 13, 5, 4, 2, 2
8	−71,732	143,532	143,753	143,645	0.001	0.830	53, 21, 11, 6, 4, 2, 2, 1
9	−71,695	143,466	143,714	143,593	0.058	0.810	50, 21, 10, 6, 5, 4, 2, 1, 1
10	−71,660	143,404	143,677	143,544	0.045	0.815	49, 21, 10, 6, 5, 4, 2, 1, 1, 1
Age 14							
1	−71,687	143,386	143,425	143,406	-	-	100
2	−70,555	141,131	141,196	141,164	0.000	0.939	91, 09
3	−69,543	139,114	139,205	139,160	0.000	0.922	80, 11, 9
4	−69,228	138,493	138,610	138,553	0.047	0.930	79, 11, 7, 3
5	−69,020	138,085	138,228	138,158	0.135	0.933	78, 11, 7, 2, 2
6	−68,847	137,746	137,915	137,832	0.050	0.940	75, 12, 7, 3, 2, 1
7	−68,742	137,545	137,740	137,645	0.041	0.925	74, 7, 7, 6, 3, 2, 1
8	−68,627	137,323	137,545	137,437	0.214	0.921	72, 7, 7, 5, 3, 3, 2, 1
9	−68,518	137,112	137,359	137,238	0.104	0.922	71, 7, 7, 5, 3, 3, 3, 1, <1
10	−68,420	136,924	137,197	137,064	0.331	0.924	71, 7, 6, 5, 3, 3, 3, 1, 1, <1
Age 16							
1	−66,872	133,756	133,795	133,776	-	-	100
2	−65,418	130,856	130,921	130,890	0.000	0.986	97, 3
3	−64,455	128,938	129,029	128,984	0.004	0.979	91, 5, 4
4	−63,913	127,862	127,979	127,922	0.176	0.980	89, 5, 5, 1
5	−63,630	127,305	127,448	127,378	0.691	0.978	86, 7, 4, 2, 1
6	−63,232	126,517	126,687	126,604	0.284	0.975	85, 4, 4, 4, 3, 1
7	−62,876	125,812	126,008	125,912	0.163	0.979	84, 4, 4, 4, 3, 2, <1
8	−62,581	125,231	125,452	125,344	0.696	0.978	83, 4, 4, 3, 3, 3, 2, <1
9	−62,324	124,725	124,972	124,851	0.202	0.980	82, 4, 3, 3, 3, 2, 2, 1, <1
10	−62,083	124,251	124,524	124,391	0.177	0.980	84, 4, 3, 3, 2 2, 1, 1, 1, <1

### Stage two: model modifications

Regular (invariant and non-invariant) LTA models were estimated by incorporating all latent profile analyses in a single 3 × 3 design (i.e., three profiles across three timepoints). This process was repeated for models including random intercepts such that invariant and non-invariant RI-LTA models, also with a 3 × 3 design were fit to the data (Table 2).

**Table 2:** Model fit of invariant and non-invariant regular LTA and RI-LTA models.

Model	BIC	Satorra-bentler (*p*)	Entropy	Class proportions based on estimated posterior probabilities (%)
3x3	416762	-	Overall =.853	
Regular LTA			T1 =.739	T1 = 60, 39, 1
(invariant)			T2 =.848	T2 = 72, 18, 10
			T3 =.963	T3 = 92, 6, 2
3x3	415442	-	Overall =.850	
RI-LTA			T1 =.758	T1 = 63, 37, <1
(invariant)			T2 =.822	T2 = 73, 17, 10
			T3 =.956	T3 = 92, 6, 2
3x3	410900	<.050	Overall =.901	
Regular LTA			T1 =.806	T1 = 61, 23, 16
(non-invariant)			T2 =.918	T2 = 77, 13, 10
			T3 =.975	T3 = 89, 7, 4
3x3	410417	<.050	Overall =.902	
RI-LTA			T1 =.808	T1 = 61, 24, 15
(non-invariant)			T2 =.919	T2 = 77, 13, 10
			T3 =.975	T3 = 89, 7, 4

### Model fit of invariant and non-invariant regular LTA and RI-LTA models

**Fig. 4:**
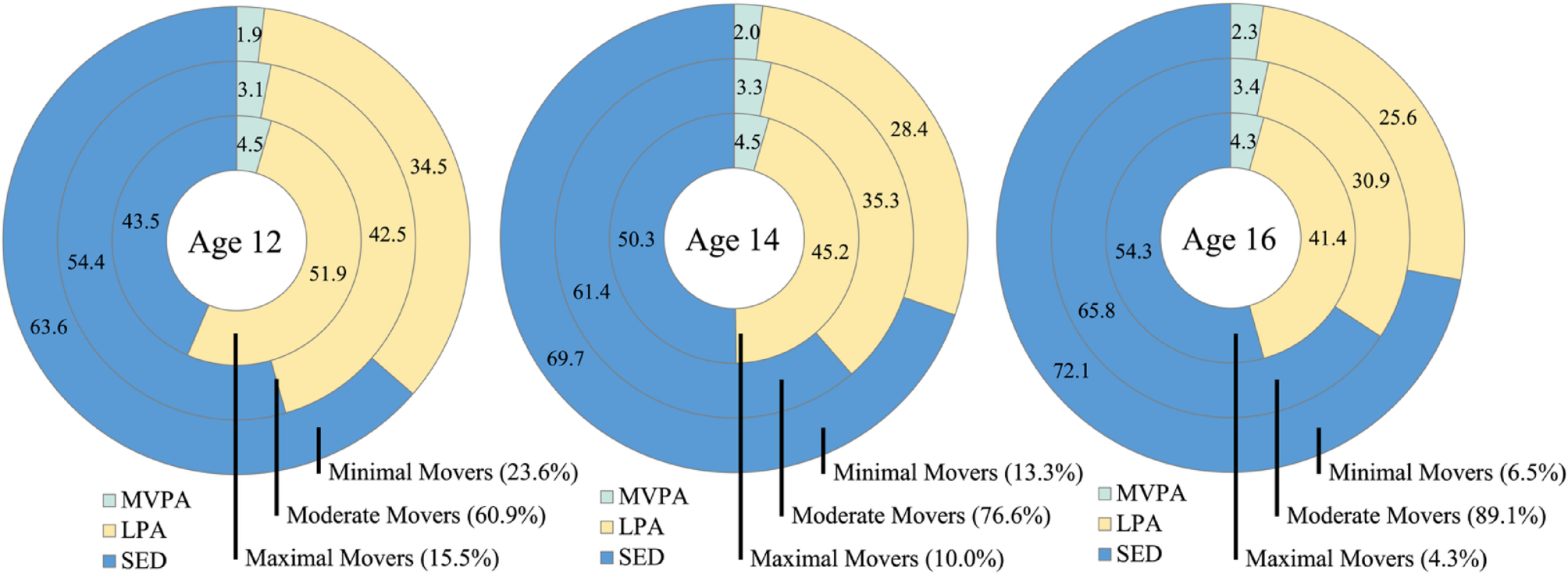
Minimally adjusted 3 × 3 non-invariant RI-LTA model, with movement behaviours expressed as the proportion of daily accelerometer wear time (%).

Invariant models contained very small classes and sample distributions while non-invariant models were more closely aligned with those established during initial profile enumeration (stage one). Highly significant Satorra-Bentler tests demonstrated measurement non-invariance for both regular LTA and RI-LTA models while BIC was lowest for the non-invariant RI-LTA model overall. Therefore, in line with recent evidence that RI-LTAs represent an advancement of the state of the art, the 3 × 3 non-invariant RI-LTA was considered the most quantitatively and qualitatively robust measurement model and was selected for advancement. Entropy for the 3 × 3 non-invariant RI-LTA overall was 0.902 (T1 = 0.808; T2 = 0.919; T3 = 0.975) signalling excellent classification accuracy. The final model is illustrated in Fig. 4. Estimated mean values for profile indicators and wear time are provided as supplementary material. In the complete case sensitivity analyses, a 3 × 3 non-invariant RI-LTA model was retained as the best fit to the data. As in the main analyses, profiles fit the label of *Maximal*, *Moderate* and *Minimal* movers and all became less active over time. However, *Maximal Movers* consistently performed slightly more MVPA and the proportional distribution was more heavily weighted toward the *Minimal Mover* profile indicating results may be somewhat sensitive to the methodological approach taken (see supplementary material for more information).

### Stage three: profile transitions and their predictors

Transition probabilities are expressed as Odds Ratios (ORs) with 95% Confidence Intervals (CIs) in Table 3. For each transition, the pattern denoting stability (i.e., the diagonal of the probability table) serves as reference. Predictors of profile transitions are reported in Table 4.

**Table 3:** Cross-tabulation indicating odds of profile transition from T1 to T2 and T2 to T3 for each latent movement profile.

	T2					
	Maximal Movers (10.0%)		Moderate Movers (76.6%)		Minimal Movers (13.4%)	
T1	*n*	OR [95%CI]	*n*	OR [95%CI]	*n*	OR [95%CI]
Maximal Movers (15.5%)	164	ref.	568	4.65 [3.76 to 5.76]^*^	10	0.05 [0.022 to 0.114]^*^
Moderate Movers (60.9%)	317	0.09 [0.08 to 0.11]^*^	2,436	ref.	316	0.07 [0.06 to 0.09]^*^
Minimal Movers (23.6%)	7	0.03 [0.01 to 0.08]^*^	823	4.25 [3.56 to 5.07]^*^	323	ref.

	T3					
	Maximal Movers (4.3%)		Moderate Movers (89.2%)		Minimal Movers (6.5%)	
T2	*n*	OR [95%CI]	*n*	OR [95%CI]	*n*	OR [95%CI]
Maximal Movers (10.0%)	68	ref.	416	13.53 [9.69 to 18.90]^*^	4	0.03 [0.01 to 0.10]^*^
Moderate Movers (76.6%)	138	0.02 [0.02, 0.03]^*^	3,505	ref.	184	0.01 [0.01 to 0.02]^*^
Minimal Movers (13.4%)	6	0.11 [0.04 to 0.29]^*^	509	15.74 [11.60 to 21.37]^*^	134	ref.

^*^ Odds adjusted for accelerometer wear time, sex, BMI, parental education and baseline depressive symptoms.

From T1 to T2, the odds of transitioning from moderate to maximal (*n* = 326, OR = 0.09 [0.08 to 0.11]) or moderate to minimal (*n* = 335, OR = 0.07 [0.06 to 0.09]) were very low compared to remaining in the same category (*n =* 2,363) indicating a high-level of stability for the moderate mover profile. Stability of the moderate mover profile was also observed across T2 to T3 (*n* = 3,480) with very low odds of transitioning from moderate to maximal (*n* = 137, OR = 0.02 [0.02 to 0.03]), or moderate to minimal (*n* = 185, OR = 0.01 [0.01 to 0.02]).

**Table 4:** Predictors of profile transitions with 95% confidence intervals.

		T2				
Sex	T1	Maximal Movers		Moderate Movers		Minimal Movers
	Maximal Movers	ref.		1.96 [1.55 to 2.48]^*^		3.68 [2.74 to 4.94]^*^
	Moderate Movers	.51 [.40 to.65]^*^		ref.		1.88 [1.49 to 2.35]^*^
	Minimal Movers	.27 [.20 to.37]^*^		0.53 [.43 to.67]^*^		ref.
		T3				
	T2	Maximal Movers		Moderate Movers		Minimal Movers
	Maximal Movers	ref.		1.34 [.97 to 1.85]		2.20 [1.44 to 3.36]^*^
	Moderate Movers	.75 [.54 to 1.03]		ref.		1.64 [1.17 to 2.31]^*^
	Minimal Movers	.46 [.30 to.70]^*^		.61 [.43 to.86]^*^		ref.
		T2				
BMI	T1	Maximal Movers		Moderate Movers		Minimal Movers
	Maximal Movers	ref.		1.04 [1.00 to 1.08]		1.08 [1.03 to 1.13]^*^
	Moderate Movers	.97 [.93 to 1.00]		ref.		1.04 [1.01 to 1.08]^*^
	Minimal Movers	.93 [.88 to.97]^*^		.96 [.93 to.99]^*^		ref.
		T3				
	T2	Maximal Movers		Moderate Movers		Minimal Movers
	Maximal Movers	ref.		1.00 [.95 to 1.05]		.96 [.90 to 1.02]
	Moderate Movers	1.00 [.96 to 1.05]		ref.		.96 [.91 to 1.01]
	Minimal Movers	1.04 [.98 to 1.11]		1.04 [.99 to 1.09]		ref.
		T2				
Parental Education	T1	Maximal Movers		Moderate Movers		Minimal Movers
	Maximal Movers	ref.		1.20 [1.09 to 1.33]^*^		1.31 [1.16 to 1.49]^*^
	Moderate Movers	.83 [.76 to.92]^*^		ref.		1.09 [.99 to 1.21]
	Minimal Movers	.76 [.67 to.86]^*^		.92 [.83 to 1.01]		ref.
		T3				
	T2	Maximal Movers		Moderate Movers		Minimal Movers
	Maximal Movers	ref.		1.09 [.94 to 1.24]		1.09[.91 to1.29]
	Moderate Movers	.93 [.81 to 1.07]		ref.		1.01[.88 to 1.16]
	Minimal Movers	.92 [.77 to 1.10]		.99 [.87 to 1.14]		ref.
		T2				
Baseline SMFQ	T1	Maximal Movers		Moderate Movers		Minimal Movers
	Maximal Movers	ref.		1.00 [.97 to 1.03]		1.01 [.98 to 1.05]
	Moderate Movers	1.00 [.97 to 1.03]		ref.		1.01 [.98 to 1.04]
	Minimal Movers	.99 [.95 to 1.03]		0.99 [.96 to 1.02]		ref.
		T3				
	T2	Maximal Movers		Moderate Movers		Minimal Movers
	Minimal Movers	ref.		1.00 [.96 to 1.05]		1.03 [.97 to 1.09]
	Moderate Movers	1.00 [.95 to 1.05]		ref.		1.03 [.98 to 1.07]
	Minimal Movers	.97 [.92 to 1.03]		.98 [.94 to 1.02]		ref.

^*^Predictor significantly contributed to the probability to profile transition.

All predictors were added to the model simultaneously.

Sex coded as 0 = male 1 = female, thus odds >1 infer greater likelihood for females.

Females were half as likely as males to transition from moderate to maximal movers from T1 to T2 (OR = 0.51 [0.40 to 0.65]) and much more likely to transition from moderate to minimal from T1 to T2 (OR = 1.88 [1.49 to 2.35]) and T2 to T3 (OR = 1.64 [1.17 to 2.31]). Higher BMI increased odds of transitioning from moderate to minimal from T1 to T2 (OR = 1.04 [1.01 to 1.08]) while higher level of parental education decreased odds of transitioning from moderate to maximal movers from T1 to T2 (OR = 0.83 [0.76 to 0.92]). Depressive symptoms reported at age 13 did not influence the odds of transitioning profiles across any timepoints. Sensitivity analyses wherein predictors were estimated sequentially in separate models produced effect sizes of equal magnitude and significance, strengthening the robustness of findings (supplementary material).

### Stage four: comparing depressive symptoms across profiles and transition probabilities

Wald difference tests comparing depressive symptoms at ages 18 and 22 for those with different transition patterns are reported in Table 5. For ease of interpretation, maximal, moderate and minimal movement profiles are denoted as 1, 2, and 3, respectively. Thus, a transition pattern of maximal at age 12, moderate at age 14 and minimal at age 16 would be expressed as 1→2→3 (see also Fig. 2 for an illustrative example). As the focus of the current study was on *profile transitions*, cross-sectional between-profile differences in depressive symptoms are not reported herein. For completeness, these analyses are provided as supplementary material.

**Table 5:** Depressive symptoms associated with transition patterns with consistently moderate movers serving as reference.

	Transition		SMFQ	Wald test statistics ^a^			SMFQ	Wald test statistics ^a^		
	Pattern	*n* (%)	at 18 (*SE*)	(*SE*)	*p*	*d*	at 22 (*SE*)	(*SE*)	*p*	*d*
Minimally Adjusted ^b^	1→1→2	139 (2.8)	4.46 (.37)	-.64 (.38)	.10	.15	2.82 (.32)	-.64 (.33)	.05	.15
	1→2→2	525 (10.6)	4.56 (.19)	-.54 (.22)^*^	.01	.13	2.61 (.16)	-.85 (.19)^*^	<.001	.20
	2→1→2	272 (5.5)	4.86 (.27)	-.24 (.29)	.40	.06	3.19 (.22)	-.27 (.25)	.28	.07
	**2→2→2**	**2,213 (44.6)**	**5.10 (.10)**	**ref.**			**3.46 (.10)**	**ref.**		
	2→3→2	252 (5.1)	6.27 (.36)	1.17 (.38)^*^	<.01	.27	4.59 (.42)	1.13 (.44)^*^	.01	.27
	3→2→2	763 (15.4)	5.55 (.18)	.45 (.22)^*^	.04	.10	3.90 (.18)	.44 (.22)^*^	.05	.11
	3→3→2	252 (5.1)	5.86 (.32)	.75 (.34)^*^	.03	.17	4.87 (.40)	1.41 (.41)^*^	<.01	.34
Fully Adjusted ^c^	1→1→2	135 (2.7)	1.56 (.51)	-.20 (.33)	.55	.05	-.11 (.50)	-.25 (.31)	.43	.06
	1→2→2	522 (10.5)	1.56 (.46)	-.19 (.21)	.38	.05	-.42 (.44)	-.56 (.18)^*^	<.01	.14
	2→1→2	278 (5.6)	1.77 (.50)	.02 (.27)	.95	.00	.12 (.47)	-.02 (.24)	.92	.01
	**2→2→2**	**2,202 (44.4)**	**1.75 (.45)**	**ref.**			**.14 (.44)**	**ref.**		
	2→3→2	255 (5.1)	2.62 (.57)	.87 (.36)^*^	.02	.21	.98 (.60)	.84 (.42)^*^	.05	.21
	3→2→2	769 (15.5)	1.92 (.49)	.17 (.21)	.43	.04	.28 (.48)	.14 (.21)	.51	.04
	3→3→2	249 (5.0)	2.65 (.57)	.62 (.33)	.06	.15	.98 (.60)	1.29 (.41)^*^	<.01	.32

1 = maximal movers; 2 = moderate movers; 3 = minimal movers.

^a^Wald tests subtracted reference group mean/intercept from comparison group mean/intercept hence, a negative value indicates comparison group had fewer symptoms, a positive value indicates comparison group had greater symptoms.

^b^adjusted for wear time during model modifications process in stage two.

^c^additionally adjusted for sex, BMI, parental education, baseline depressive symptoms.

^*^significant difference.

In all comparisons, those with a consistent moderate mover pattern (i.e., 2→2→2) were used as the reference against which all other transition patterns were compared. After adjusting for covariates, those whose prior movement levels fluctuated but contained a period of moving less often (i.e., 2→3→2 vs. 2→2→2) consistently reported significantly greater depressive symptoms at age 18 (*p* =.02, *d* = 0.21), and age 22 (*p* =.05, *d* = 0.21). The largest effect was observed for those who consistently used to move less (i.e., 3→3→2 vs. 2→2→2) with these individuals reporting significantly greater depressive symptoms at age 22 (*p* <.01, *d* = 0.32). Finally, those who moved more during early adolescence (i.e., 1→2→2 vs. 2→2→2) also reported significantly fewer depressive symptoms at age 22 (*p* <.01, *d* = 0.14).

## Discussion

The aim of this study was to establish latent profiles of movement behaviour throughout adolescence, evaluate the stability of these profiles over time, and the extent to which transitioning between profiles contributes to variance in depressive symptoms in both late adolescence and early adulthood. Three latent profiles were identified across all three timepoints differentiating between *Maximal Movers*, *Moderate Movers*, and *Minimal Movers*. MVPA and LPA decreased while sedentary behaviour increased antagonistically between and within profiles at every timepoint, in line with H^1^. Although maximal, moderate, and minimal movers were consistently identified throughout adolescence, quantitative assessment of measurement non-invariance presented statistical evidence that all profiles became more sedentary and less physically active over time, providing support for H^2^.

Findings align with extant literature suggesting that adolescent movement becomes increasingly homogeneous over time^9^; contradict evidence of the “Active Couch Potato Hypothesis” (i.e., the notion that volumes of MVPA can persist despite increases in sedentary behaviour)^56,57^; and provide evidence that convergence of movement behaviours persists even when sedentary behaviour is included as a key profile indicator, underscoring the need for a holistic approach in public health initiatives. Interventions should concurrently target improvements in PA and reductions sedentary behaviour to promote well-balanced movement profiles indicative of a healthy lifestyle. Furthermore, for initiatives to be maximally efficacious, they should target adolescents when health behaviours are most malleable^58^. Our evidence shows the earlier, the better.

Adolescents’ daily lives are predominantly sedentary and become increasingly so as they age. To foster optimal compositions of waking movement, public health guidelines should specify that increases in LPA or MVPA should specifically displace time spent sedentary, as opposed to each other. This is substantiated by evidence that displacing sedentary behaviour with MVPA yields a greater collective health benefit than if displacing LPA^24^. The centrality of school-based intervention efforts makes this challenging due to the practical constraints of integrating PA into environments that are inherently sedentary in nature. Potential scalable solutions include: “Classroom Movement Breaks” – structured 5–10 minute lesson breaks for stretching and dynamic movements; and “Physically Active Learning” – practices that require pupils to move around the classroom to complete tasks, both of which are promising for enhancing movement volume, academic performance and wellbeing^59^. There is also evidence among younger children (aged < 10) that integrating a ‘Daily Mile’ into everyday school routines improves visual spatial working memory and physical fitness^60^. However, fostering an autonomously motivating PA experience that ensures reliable sustainability of such significant changes in children’s behaviour remains an elusive goal for public health, requiring increased application of behaviour change theory science^61^. Females were more likely to transition to profiles characterised by less PA and greater sedentary behaviour than males, in line with H^3a^ however, differences were more pronounced from T1 (age 12) to T2 (age 14) than from T2 (age 14) to T3 (age 16). Higher BMI increased odds, while higher parental education decreased odds, of transitioning to profiles that ultimately moved less, but differences were only observed from T1 to T2 and not T2 to T3. Therefore, findings offer partial support for H^3b^ and contradictory evidence against H^3c^.

From a mental health perspective, sedentary behaviour serves as a risk factor in some instances, but can also be supportive through activities such as reading for enjoyment and engagement in artistic hobbies^17^. Nonetheless, the minimal movement inherent in these behaviours is inconsistent with the promotion of optimal *physical* health. Accordingly, early intervention is warranted, particularly among adolescent females, who report higher levels of screen-time compared to males and exhibit greater attrition from physically active pursuits such as organised sports^62^. While female drop-off in PA and drop-out from sport is a pervasive issue, initiatives that successfully sustain engagement or promote re-engagement could support wellbeing by leveraging the organic social support networks that exist within sporting environments. Intervention developers should therefore be cognisant that many young females express a preference for non-competitive PA settings that facilitate social interaction, and frequently cite a lack of enjoyment or intrinsic motivation to participate as antecedents of dropout^63,64^.

Depressive symptoms at the outset of adolescence did not predict subsequent changes in movement behaviour. Although contrary to our hypothesis [H^3d^], this is not surprising given initial onset of depressive symptoms typically occurs at 15.5 years of age^2^. While individual variability makes it likely *some* individuals will have experienced severe symptoms at age 13, for the sample collectively, symptoms at this time were inconsequential for future profile transitions. Future research should explore if depressive symptoms lead to transitions in movement when measured at the average age of onset (i.e., slightly later in adolescence) to support development of age-sensitive interventions.

Fully adjusted models provided evidence that compared to consistently moderate movers, those who previously endorsed greater (a.k.a., maximal) levels of overall daily movement in early adolescence (1→2→2 vs. 2→2→2) reported comparatively fewer depressive symptoms at age 22 (*p* <.01; *d* = 0.14) but not at age 18 (*p* =.38; *d* = 0.05) aligning with H^4b^ but not H^4a^. Those demonstrating fluctuating movement patterns containing a period of less frequent movement (2→3→2 vs. 2→2→2) reported more depressive symptoms at age 18 (*p* =.02; *d* = 0.21); and age 22 (*p* <.05; *d* = 0.21) supporting H^5a^ and H^5b^, respectively. Lastly, the largest effect on depressive symptoms was observed for those who had been consistently minimal movers in the past compared to those consistently moderate at all three timepoints (3→3→2 vs. 2→2→2) who reported significantly greater symptoms at age 22 (*p* <.01; *d* = 0.32) but not at age 18 (*p* =.06; *d* = 0.15) providing further support for H^5b^ but not H^5a^.

Findings allude to the importance of often and consistently throughout adolescence for health promotion^65^ and that stability in movement behaviour may protect against depressive symptoms in late adolescence and early adulthood. The Overflow Hypothesis^27^ posits consistent movement levels may be indicative of a well-adjusted lifestyle and coincide with regular participation in other mentally-supportive health behaviours. Physically active adolescents, for example, have been shown to report higher sleep quality and healthier eating habits than their less active peers^10^. A consistently well adapted composition of movement behaviours, therefore, may lay a stable foundation for wellbeing that transcends the immediate effects of movement behaviour alone, extending to a constellation of health behaviours that collectively help to mitigate depressive symptoms.

Consistent movement also supports regulation of the hypothalamic-pituitary-adrenal axis, the body’s primary biological stress response system, thereby stabilising cortisol levels and fostering long-term psychological support^66^. Intermittent volumes of movement on the other hand, may not facilitate the same level of regulatory adaptation. Rather, fluctuating movement patterns, specifically those that include periods of minimal movement, may reflect broader lifestyle instability linked to greater psychological distress^28^. Substantial increases in daily movement are also likely to coincide with a period of lifestyle adjustment, presenting challenges (e.g., fatigue; disruption to daily routines) the immediate effects of which on balance, may outweigh the benefits in the short-term.

The benefits of moving more at age 16 may not fully counterbalance the adverse effects of prolonged periods of minimal movement earlier in life (3→3→2). Conversely, high levels of movement when aged 12 followed by prolonged moderate movement (1→2→2) could significantly reduce depressive symptoms at age 22 compared to moving moderately throughout. Findings emphasise the importance of early intervention and moving often when young^58^ and may stem from a broad range of causal pathways including improved neurological development^67^, identity formation and self-concept^68^, and social support networks established through participation in PA^69^. These proposed pathways may instil long-lasting psychological resources and habits that protect against depressive symptoms long-term^5,70,71^.

The current study has a number of limitations that must be born in mind. First, it did not explore the context within which movement behaviours were practised^72^. Exploration of psychological, social, and environmental influences surrounding movement behaviours would facilitate more nuanced, targeted public health strategies for improving adolescent mental and physical health^73^. Other potential sources of confounding include additional sociodemographic factors (e.g., ethnicity, special educational needs), perceptions of social support, pre-existing health conditions, and prior intervention exposure. Second, emerging studies using *Compositional Data Analysis* (CoDA) to assess daily movement, often incorporate sleep to establish 24-hour movement profiles^24,74^. Device-based sleep data were not available for this study, limiting analysis to *waking* movement behaviours. Given that non-wear time is predominantly comprised of time spent asleep, we contend that adjustment for wear time in both partially and fully adjusted models indirectly controls for variability in sleep duration—alongside other, less frequent causes of non-wear. While this limitation was inherent to the study design, future investigations should seek to incorporate sleep either as a potential confounder or as a key component of the 24-hour behavioural continuum. From a methodological standpoint, latent profiles were derived using a numerical constant (100% of accelerometer wear time)^24,74^. Although not an explicit application of CoDA, this approach adheres to its core principles and remains consistent with current methodological standards. Third, depressive symptoms were quantified using self-reports as opposed to clinical assessment. Sceptics query the reliability of young peoples’ self-reported symptoms due unavoidable subjectivity, and the risk of reporter- and social desirability bias. While evidence suggests these risks can be minimised by restricting analysis to the use of composite scores as opposed to designated thresholds above which scores are said to represent elevated or ‘clinically relevant’ symptoms^75^, care should be taken in interpretation.

Distinct profiles of movement behaviour exist throughout adolescence. Most individuals exhibit moderate levels of movement at age 12 or shift to moderate levels later as they progress through adolescence, although what constitutes ‘moderate movement’ incrementally declines as all adolescents moved less over time. Females, those with higher BMI, and with more educated parents were at greater risk of early transition to profiles marked by reduced PA and increased sedentary behaviour. Moving often when age 12 led to significantly fewer depressive symptoms in early adulthood, even after transitioning to a prolonged period of moderate movement. Collectively, findings highlight early adolescence as a critical period for the delivery of intervention/prevention strategies designed to either initiate or maintain healthy patterns of movement behaviour and mitigate future depressive symptoms. Such approaches may benefit from adopting evidence-based contemporary models of behaviour change that focus on nurturing individuals’ psychological needs, along with strategies that are attentive to adolescents’ social circumstances and cultural context, to ultimately facilitate autonomously motivating and lasting behaviour change.

## Electronic supplementary material

Below is the link to the electronic supplementary material.

Supplementary Material 1

## Data Availability

The data that support the findings of this study are available from the ALSPAC Executive but restrictions apply to the availability of these data, which were used under license for the current study, and so are not publicly available. Data are however available from the ALSPAC Executive upon submission of a research proposal through the ALSPAC website (https://www.bristol.ac.uk/alspac/researchers/access/) where researchers can also access a data dictionary, variable catalogue and variable search tool.
