# Supplementary material for: Stability and change in latent movement behaviour profiles during adolescence and links with future depressive symptoms
Source: Sci Rep. 2025 Jul 1;15:21716. doi: 10.1038/s41598-025-04466-7 (PMC12217875; doi:10.1038/s41598-025-04466-7)

**Moving Through Time: Stability and Change in Adolescent Movement Behaviour and links with Future Depressive Symptoms
(Supplementary Materials)**

Table of Contents

[Supplementary Material for the Main Analytical Model 3](#_Toc198796302)

[Invariance Testing: Satorra-Bentler scaled (mean-adjusted) chi-square difference test 4](#_Toc198796303)

[Comparison of Regular LTA models: 5](#_Toc198796304)

[Comparison of RI-LTA models: 5](#_Toc198796305)

[Estimated Means and Sample Proportions for each Latent Profile 6](#_Toc198796306)

[Predictors of Profile Transitions: Effects of Each Predictor Tested Independently in Separate Models 7](#_Toc198796307)

[Differences in Depressive Symptoms at Age 18 and 22 according to Profile Membership at T3 8](#_Toc198796308)

[Sensitivity Analysis: Equivalent Models that do not Adjust for Accelerometer Wear Time 9](#_Toc198796309)

[Invariance Testing: Satorra-Bentler scaled (mean-adjusted) chi-square difference test 10](#_Toc198796310)

[Comparison of Regular LTA models: 10](#_Toc198796311)

[Comparison of RI-LTA models: 10](#_Toc198796312)

[Fit of Invariant and Non-Invariant Regular LTA and RI-LTA models: 11](#_Toc198796313)

[Estimated Means and Sample Proportions for each Latent Profile 12](#_Toc198796314)

[Odds of Profile Transition 13](#_Toc198796315)

[Predictors of Profile Transitions: Effects of Each Predictor Tested Simultaneously 14](#_Toc198796316)

[Predictors of Profile Transitions: Effects of Each Predictor Tested Independently in Separate Models 15](#_Toc198796317)

[Differences in Depressive Symptoms at Age 18 and 22 according to Profile Membership at T3 16](#_Toc198796318)

[Differences in Depressive Symptoms at Age 18 and 22 according to Latent Transition Probabilities 17](#_Toc198796319)

[Sensitivity Analysis: Complete Case (Attrition) Analysis 18](#_Toc198796320)

[Model Fit Indices for Cross-Sectional Latent Profiles of Adolescent Movement Behaviour Controlling for Wear Time (Complete Case) 19](#_Toc198796321)

[Elbow Plots Illustrating Model Fit of Cross-Sectional Latent Profile Models with k+1 Solutions (Complete Case) 20](#_Toc198796322)

[Invariance Testing: Satorra-Bentler Scaled (Mean-Adjusted) Chi-Square Difference Test 21](#_Toc198796323)

[Comparison of Regular LTA models: 21](#_Toc198796324)

[Comparison of RI-LTA models: 21](#_Toc198796325)

[Fit of Invariant and Non-Invariant Regular LTA and RI-LTA Models (Complete Case) 22](#_Toc198796326)

[Minimally Adjusted 3x3 Non-Invariant RI-LTA Model, with Movement Behaviours Expressed as the Proportion of Daily Accelerometer Wear Time (Complete Case; %) 22](#_Toc198796327)

# Supplementary Material for the Main Analytical Model

## Invariance Testing: Satorra-Bentler scaled (mean-adjusted) chi-square difference test

Retrieved from: <https://www.statmodel.com/chidiff.shtml>

A model with full measurement invariance is nested within one where measurement parameters are freely estimated. The fit of the two models can be compared using a *Likelihood Ratio Test* (LRT). By default, Mplus uses the MLR estimator which requires a correction when calculating a LRT between two models. Steps taken to compute a Satorra-Bentler scaled (mean-adjusted) chi-square difference test based on loglikelihood values and scaling correction factors obtained with the MLR estimator were as follows. The nested model H^0^ is the more restrictive (invariant) model with fewer degrees of freedom than the comparison (non-invariant) model H^1^.

L_0_  = Log-Likelihood of the nested (null) model (that with equality constraints; i.e. invariant model);

L_1_  = Log-Likelihood of the comparison model without the added constraints (i.e. non-invariant model);

c_0_  = Scaling correction factor of the nested (null) model (that with equality constraints; invariant);

c_1_  = Scaling correction factor of the comparison model without the added constraints (non-invariant);

p_0_  = Free parameters in the nested (null) model (that with equality constraints; invariant);

p_1_  = Free parameters in the comparison model without the added constraints (non-invariant);

1. Estimate the nested and comparison models in Mplus using MLR. The output provides loglikelihood values L^0^ and L^1^ and scaling correction factors c^0^ and c^1^ for the H^0^ and H^1^ models:

|  | Regular LTA models | RI-LTA models |
| --- | --- | --- |
| L^0^ | -208219.250 | -207546.701 |
| c^0^ | 1.6810 | 1.5679 |
| p^0^ | 38 | 41 |
| L^1^ | -205211.907 | -204957.543 |
| c^1^ | 2.1371 | 2.2300 |
| p^1^ | 56 | 59 |

1. Formula used to compute the difference test scaling correction where p^0^ is the number of parameters in the nested model and p^1^ is the number of parameters in the comparison model.

cd = (p^0^ * c^0^ - p^1^*c^1^)/(p^0^ - p^1^)

TRd = -2*(L^0^ - L^1^)/cd

df = p1 – p0

1. Compare result against a chi square distribution table for the number of df where *p* < .05

Table found at: <https://www.medcalc.org/manual/chi-square-table.php>

### Comparison of Regular LTA models:

cd = (38 * 1.6810 – 56 * 2.1371)/(38 - 56) = 3.100

TRd = -2*(-208219.250 + 205211.907)/3.100 = 1940.221

df = 56 – 38 = 18

The test result of 1940.221 exceeds the critical value on a chi-square distribution for *df*=18 (22.869) meaning the test result is highly significant, suggesting the non-invariant (comparison) model yields a significantly better fit to the data than the invariant (nested) model. The null hypothesis of invariance was rejected, and parameters were allowed to vary over time.

### Comparison of RI-LTA models:

cd = (41 * 1.5679 – 59 * 2.2300)/(41 - 59) = 3.738

TRd = -2*(-207546.701 + 206345.720)/3.738 = 642.579

df = 59 – 41 = 18

The test result of 642.579 exceeds the critical value on a chi-square distribution for *df*=18 (22.869) meaning the test result is highly significant, suggesting that the non-invariant (comparison) model yields a significantly better fit to the data than the invariant (nested) model. The null hypothesis of invariance was rejected, and parameters were allowed to vary over time.

Highly significant Satorra-Bentler LRTs indicated that non-invariance was also observed in the RI-LTA models and quantitative fit statistics unanimously favoured the non-invariant RI-LTA model. When considering the proportional distribution of the sample, invariant models contained very small classes (regular LTA T1 = 1%; regular LTA T3 2%; RI-LTA T1 <1%; RI-LTA T3 = 2%) thus, were considered unstable with limited generalisability to populations beyond that immediately under investigation. Conversely, proportional distributions of the sample in non-invariant models were more closely aligned to those identified when profiles at each timepoint were enumerated separately. Therefore, in line with recent evidence that RI-LTAs represent an advancement of the state of the art, the 3x3 non-invariant RI-LTA was considered the most quantitatively and qualitatively robust measurement model and was selected for advancement. Entropy for the 3x3 non-invariant RI-LTA was .902 signalling excellent classification accuracy.

## Estimated Means and Sample Proportions for each Latent Profile

| Age 12 | | | | | |
| --- | --- | --- | --- | --- | --- |
|  | Unit | Maximal Movers | Moderate Movers | Minimal Movers |  |
| Count | n (%) | 767 (15.5) | 3,024 (60.9) | 1,173 (23.6) |  |
| Wear time | Mean (SE) | 779.94 | 778.00 | 787.50 |  |
| MVPA minutes | Mean (SE) | 35.33 (1.03) | 24.07 (.27) | 15.11 (.35) |  |
| (proportion of wear time) | % | 4.5 | 3.1 | 1.9 |  |
| LPA minutes | Mean (SE) | 405.02 (2.31) | 330.43 (.99) | 271.26 (1.79) |  |
| (proportion of wear time) | % | 51.9 | 42.50 | 34.4 |  |
| Sedentary minutes | Mean (SE) | 339.59 (2.60) | 423.47 (1.12) | 501.09 (1.72) |  |
| (proportion of wear time) | % | 43.5 | 54.4 | 63.5 |  |
| Age 14 | | | | | |
|  | Unit | Maximal Movers | Moderate Movers | Minimal Movers |  |
| Count | n (%) | 499 (10.0) | 3,801 (76.6) | 664 (13.4) |  |
| Wear time | Mean (SE) | 797.60 | 789.50 | 810.20 |  |
| MVPA minutes | Mean (SE) | 36.08 (1.30) | 26.28 (.18) | 15.90 (.50) |  |
| (proportion of wear time) | % | 4.5 | 3.3 | 2.0 |  |
| LPA minutes | Mean (SE) | 360.71 (2.51) | 278.66 (.45) | 229.66 (2.19) |  |
| (proportion of wear time) | % | 45.2 | 35.3 | 28.3 |  |
| Sedentary minutes | Mean (SE) | 400.80 (2.98) | 484.55 (.49) | 564.58 (2.15) |  |
| (proportion of wear time) | % | 50.3 | 61.4 | 69.7 |  |
| Age 16 | | | | | |
|  | Unit | Maximal Movers | Moderate Movers | Minimal Movers |  |
| Count | n (%) | 213 (4.3) | 4,427 (89.2) | 324 (6.5) |  |
| Wear time | Mean (SE) | 781.59 | 796.17 | 829.48 |  |
| MVPA minutes | Mean (SE) | 33.65 (1.97) | 27.00 (.14) | 18.65 (1.03) |  |
| (proportion of wear time) | % | 4.3 | 3.4 | 2.3 |  |
| LPA minutes | Mean (SE) | 323.57 (7.40) | 245.62 (.31) | 212.54 (2.77) |  |
| (proportion of wear time) | % | 41.4 | 30.9 | 25.6 |  |
| Sedentary minutes | Mean (SE) | 424.37 (5.90) | 523.55 (.34) | 598.29 (3.10) |  |
| (proportion of wear time) | % | 54.3 | 65.8 | 72.1 |  |
| Sex | n (% female) | 87 (40.8) | 2,315 (52.3) | 206 (63.6) |  |
| BMI | Mean (SE) | 19.64 (.21) | 19.75 (.05) | 19.58 (.21) |  |
| Parental Education | Mean (SE) | 2.82 (.08) | 2.89 (.02) | 3.15 (.07) |  |

## Predictors of Profile Transitions: Effects of Each Predictor Tested Independently in Separate Models

|  |  | Wave 2 | | |
| --- | --- | --- | --- | --- |
| Sex | Wave 1 | Maximal Movers | Moderate Movers | Minimal Movers |
|  | Maximal Movers | ref. | 1.970 (1.560,2.488)^*^ | 3.748 (2.799,5.018)^*^ |
|  | Moderate Movers | 0.508 (0.402,0.641)^*^ | ref. | 1.903 (1.518,2.385)^*^ |
|  | Minimal Movers | 0.267 (0.199,0.357)^*^ | 0.526 (0.419,0.659)^*^ | ref. |
|  |  | Wave 3 | | |
|  | Wave 2 | Maximal Movers | Moderate Movers | Minimal Movers |
|  | Maximal Movers | ref. | 1.345 (0.977,1.852) | 2.220 (1.461,3.373)^*^ |
|  | Moderate Movers | 0.743 (0.540,1.024) | ref. | 1.650 (1.177,2.313)^*^ |
|  | Minimal Movers | 0.450 (0.296,0.685)^*^ | 0.606 (0.432,0.849)^*^ | ref. |
|  |  | Wave 2 | | |
| BMI | Wave 1 | Maximal Movers | Moderate Movers | Minimal Movers |
|  | Maximal Movers | ref. | 1.038 (0.999,1.078) | 1.082 (1.035,1.131)^*^ |
|  | Moderate Movers | 0.964 (0.928,1.001) | ref. | 1.043 (1.010,1.077)^*^ |
|  | Minimal Movers | 0.924 (0.884,0.966)^*^ | 0.959 (0.929,0.990)^*^ | ref. |
|  |  | Wave 3 | | |
|  | Wave 2 | Maximal Movers | Moderate Movers | Minimal Movers |
|  | Maximal Movers | ref. | 0.997 (0.951,1.046) | 0.966 (0.908,1.027) |
|  | Moderate Movers | 1.003 (0.956,1.052) | ref. | 0.969 (0.922,1.018) |
|  | Minimal Movers | 1.035 (0.974,1.101) | 1.032 (0.982,1.085) | ref. |
| Parental |  | Wave 2 | | |
| Education | Wave 1 | Maximal Movers | Moderate Movers | Minimal Movers |
|  | Maximal Movers | ref. | 1.189 (1.080,1.310)^*^ | 1.290 (1.141,1.459)^*^ |
|  | Moderate Movers | 0.841 (0.764,0.926)^*^ | ref. | 1.085 (0.983,1.198) |
|  | Minimal Movers | 0.775 (0.685,0.876)^*^ | 0.922 (0.835,1.017) | ref. |
|  |  | Wave 3 | | |
|  | Wave 2 | Maximal Movers | Moderate Movers | Minimal Movers |
|  | Maximal Movers | ref. | 1.081 (0.941,1.241) | 1.112 (0.937,1.321) |
|  | Moderate Movers | 0.925 (0.806,1.062) | ref. | 1.029 (0.899,1.177) |
|  | Minimal Movers | 0.899 (0.757,1.068) | 0.972 (0.849,1.112) | ref. |
| Baseline |  | Wave 2 | | |
| SMFQ | Wave 1 | Maximal Movers | Moderate Movers | Minimal Movers |
|  | Maximal Movers | ref. | 1.009 (0.978,1.041) | 1.030 (0.992,1.070) |
|  | Moderate Movers | 0.991 (0.961,1.022) | ref. | 1.021 (0.992,1.051) |
|  | Minimal Movers | 0.971 (0.935,1.008) | 0.980 (0.952,1.008) | ref. |
|  |  | Wave 3 | | |
|  | Wave 2 | Maximal Movers | Moderate Movers | Minimal Movers |
|  | Maximal Movers | ref. | 1.008 (0.963,1.055) | 1.042 (0.986,1.101) |
|  | Moderate Movers | 0.992 (0.948,1.039) | ref. | 1.034 (0.992,1.078) |
|  | Minimal Movers | 0.960 (0.908,1.014) | 0.967 (0.928,1.009) | ref. |

^*^*significantly predicted transition probability*

## Differences in Depressive Symptoms at Age 18 and 22 according to Profile Membership at T3

|  |  |  | SMFQ | Wald Test ^a^ |  |  | SMFQ | Wald Test ^a^ |  |  |
| --- | --- | --- | --- | --- | --- | --- | --- | --- | --- | --- |
|  | T3 Profile | N (%) | at 18 (*SE*) | (*SE*) | *p* | *d* | at 22 (*SE*) | (SE) | *p* | *d* |
| Minimally | Minimal | 326 (6.6) | 5.90 (.29) | ref. |  |  | 4.73 (.28) | ref. |  |  |
| Adjusted ^b^ | Moderate | 4,426 (89.2) | 5.19 (.07) | -.70 (.30)^*^ | .02 | .16 | 3.54 (.06) | -1.19 (.29)^*^ | <.001 | .28 |
|  | Maximal | 211 (4.2) | 4.95 (.29) | -.94 (.41)^*^ | .02 | .22 | 3.37 (.31) | -1.37 (.42)^*^ | <.01 | .32 |
|  |  |  |  |  |  |  |  |  |  |  |
|  | Moderate |  |  | ref. |  |  |  | ref. |  |  |
|  | Maximal |  |  | -.24 (.29) | .41 | .06 |  | -.18 (.32) | .58 | .04 |
| Fully | Minimal | 326 (6.6) | 5.91 (.29) | ref. |  |  | 4.77 (.29) | ref. |  |  |
| Adjusted ^c^ | Moderate | 4,426 (89.2) | 5.19 (.07) | -.72 (.30) | .02 | .16 | 3.54 (.06) | -1.23 (.30)^*^ | <.001 | .29 |
|  | Maximal | 211 (4.2) | 4.95 (.29) | -.96 (.41) | .02 | .22 | 3.36 (.31) | -1.41 (.43)^*^ | <.01 | .33 |
|  |  |  |  |  |  |  |  |  |  |  |
|  | Moderate |  |  | ref. |  |  |  | ref. |  |  |
|  | Maximal |  |  | -.24 (.30) | .41 | .00 |  | .18 (.32) | .57 | .04 |

*^a^ Wald tests subtracted reference group mean from comparison group mean hence, a negative value indicates comparison group had fewer symptoms, a positive value indicates comparison group had greater symptoms*

*^b^ adjusted for wear time during model modifications process in stage two*

*^c^ additionally adjusted for sex, BMI, parental education, baseline depressive symptoms*

*^*^significant difference*

# Sensitivity Analysis: Equivalent Models that do not Adjust for Accelerometer Wear Time

## Invariance Testing: Satorra-Bentler scaled (mean-adjusted) chi-square difference test

1. Estimate the nested and comparison models in Mplus using MLR. The output provides loglikelihood values L^0^ and L^1^ and scaling correction factors c^0^ and c^1^ for the H^0^ and H^1^ models:

|  | Regular LTA models | RI-LTA models |
| --- | --- | --- |
| L^0^ | -208976.222 | -208238.376 |
| c^0^ | 1.6429 | 1.6609 |
| p^0^ | 32 | 35 |
| L^1^ | -206162.528 | -205911.510 |
| c^1^ | 2.1994 | 2.2815 |
| p^1^ | 50 | 53 |

1. Formula used to compute the difference test scaling correction where p^0^ is the number of parameters in the nested model and p^1^ is the number of parameters in the comparison model.

cd = (p^0^ * c^0^ - p^1^*c^1^)/(p^0^ - p^1^)

TRd = -2*(L^0^ - L^1^)/cd

df = p1 – p0

1. Compare result against a chi square distribution table for the number of df where *p* < .05

Table found at: <https://www.medcalc.org/manual/chi-square-table.php>

### Comparison of Regular LTA models:

cd = (32 * 1.6429 – 50 * 2.1994)/(32 - 50) = 3.189

TRd = -2*(-208976.222 + 206162.528)/3.189 = 1764.625

df = 50 – 32 = 18

The test result of 1764.625 exceeds the critical value on a chi-square distribution for *df*=18 (22.869) meaning the test result is highly significant, suggesting the non-invariant (comparison) model yields a significantly better fit to the data than the invariant (nested) model. The null hypothesis of invariance was rejected, and parameters were allowed to vary over time.

### Comparison of RI-LTA models:

cd = (35 * 1.6609 – 53 * 2.2815)/(35 - 53) = 3.477

TRd = -2*(-208238.376 + 205911.510)/3.477 = 1338.34

df = 53 – 35 = 18

The test result of 1338.34 exceeds the critical value on a chi-square distribution for *df*=18 (22.869) meaning the test result is highly significant, suggesting that the non-invariant (comparison) model yields a significantly better fit to the data than the invariant (nested) model. The null hypothesis of invariance was rejected, and parameters were allowed to vary over time.

## Fit of Invariant and Non-Invariant Regular LTA and RI-LTA models:

| Model | BIC | Satorra-Bentler  (*p*) | Entropy | Estimated Class Proportions Based on Posterior Probabilities (%) |
| --- | --- | --- | --- | --- |
| Regular LTA | 418224 | - | Overall = .839 |  |
| (invariant) |  |  | T1 = .738 | T1 = 56, 43, <1 |
|  |  |  | T2 = .808 | T2 = 74, 17, 9 |
|  |  |  | T3 = .956 | T3 = 93, 5, 2 |
| RI-LTA | 416774 | - | Overall = .827 |  |
| (invariant) |  |  | T1 = .744 | T1 = 61, 39, <1 |
|  |  |  | T2 = .772 | T2 = 76, 16, 8 |
|  |  |  | T3 = .946 | T3 = 94, 4, 2 |
| Regular LTA | 412750 | < .05 | Overall = .902 |  |
| (non-invariant) |  |  | T1 = .805 | T1 = 62, 22, 16 |
|  |  |  | T2 = .921 | T2 = 79, 16, 9 |
|  |  |  | T3 = .979 | T3 = 91, 5, 4 |
| RI-LTA | 412274 | < .05 | Overall = .902 |  |
| (non-invariant) |  |  | T1 = .806 | T1 = 62, 22, 16 |
|  |  |  | T2 = .921 | T2 = 79, 12, 9 |
|  |  |  | T3 = .979 | T3 = 91, 5, 4 |

Highly significant Satorra-Bentler LRTs indicated that non-invariance was also observed in the RI-LTA models and quantitative fit statistics unanimously favoured the non-invariant RI-LTA model. When considering the proportional distribution of the sample, invariant models contained very small classes (regular LTA T1 = 1%; regular LTA T3 2%; RI-LTA T1 <1%; RI-LTA T3 = 2%) thus, were considered unstable with limited generalisability to populations beyond that immediately under investigation. Conversely, proportional distributions of the sample in non-invariant models were more closely aligned to those identified when profiles at each timepoint were enumerated separately. Therefore, in line with recent evidence that RI-LTAs represent an advancement of the state of the art, the 3x3 non-invariant RI-LTA was considered the most quantitatively and qualitatively robust measurement model and was selected for advancement. Entropy for the 3x3 non-invariant RI-LTA was .902 signalling excellent classification accuracy.

## Estimated Means and Sample Proportions for each Latent Profile

| Age 12 | | | | | |
| --- | --- | --- | --- | --- | --- |
|  | Unit | Maximal Movers | Moderate Movers | Minimal Movers |  |
| Count | n (%) | 772 (15.5) | 3,086 (62.2) | 1,106 (22.3) |  |
| Wear time | Mean (SE) | 779.94 | 777.97 | 787.46 |  |
| MVPA minutes | Mean (SE) | 35.33 | 24.07 | 15.11 |  |
| (proportion of wear time) | % | 4.5 | 3.1 | 1.9 |  |
| LPA minutes | Mean (SE) | 405.02 | 330.43 | 271.26 |  |
| (proportion of wear time) | % | 51.9 | 42.5 | 34.4 |  |
| Sedentary minutes | Mean (SE) | 339.59 | 423.47 | 501.09 |  |
| (proportion of wear time) | % | 43.5 | 54.4 | 63.6 |  |
| Age 14 | | | | | |
|  | Unit | Maximal Movers | Moderate Movers | Minimal Movers |  |
| Count | n (%) | 457 (9.2) | 3,933 (79.2) | 573 (11.5) |  |
| Wear time | Mean (SE) | 797.59 | 789.49 | 810.14 |  |
| MVPA minutes | Mean (SE) | 36.08 | 26.28 | 15.90 |  |
| (proportion of wear time) | % | 4.5 | 3.3 | 2.0 |  |
| LPA minutes | Mean (SE) | 360.71 | 278.66 | 229.66 |  |
| (proportion of wear time) | % | 45.2 | 35.3 | 28.3 |  |
| Sedentary minutes | Mean (SE) | 400.80 | 484.55 | 564.58 |  |
| (proportion of wear time) | % | 50.3 | 61.4 | 69.7 |  |
| Age 16 | | | | | |
|  | Unit | Maximal Movers | Moderate Movers | Minimal Movers |  |
| Count | n (%) | 193 (3.9) | 4,512 (90.9) | 258 (5.2) |  |
| Wear time | Mean (SE) | 781.59 | 796.17 | 829.48 |  |
| MVPA minutes | Mean (SE) | 33.65 | 27.00 | 18.65 |  |
| (proportion of wear time) | % | 4.3 | 3.4 | 2.2 |  |
| LPA minutes | Mean (SE) | 323.57 | 245.62 | 212.54 |  |
| (proportion of wear time) | % | 41.4 | 30.9 | 25.6 |  |
| Sedentary minutes | Mean (SE) | 424.37 | 523.55 | 598.29 |  |
| (proportion of wear time) | % | 54.3 | 65.8 | 72.1 |  |
| Sex | n (% female) | 75 (38.9) | 2,360 (52.3) | 168 (65.1) |  |
| BMI | Mean (SE) | 19.68 (.24) | 19.74 (.05) | 19.53 (.22) |  |
| Parental Education | Mean (SE) | 2.78 (.09) | 2.90 (.02) | 3.11 (.08) |  |

## Odds of Profile Transition

| Wave 1 | Wave 2 OR [95% CI] | | |
| --- | --- | --- | --- |
| Sample Proportion (%) | Maximal Movers (9.7) | Moderate Movers (77.6) | Minimal Movers (12.6) |
| Maximal Movers (15.7) | ref. | 6.17 [4.88 to 7.80] ^*^ | .06 [.02 to .15] ^*^ |
| Moderate Movers (61.6) | .08 [.06 to .09] ^*^ | ref. | .07 [.06 to .09] ^*^ |
| Minimal Movers (22.6) | .02 [.01 to .09] ^*^ | 4.67 [3.86 to 5.63] ^*^ | ref. |
| Wave 2 | Wave 3 OR [95% CI] | | |
| Sample Proportion (%) | Maximal Movers (4.0) | Moderate Movers (90.0) | Minimal Movers (6.0) |
| Maximal Movers (9.7) | ref. | 14.50 [10.29 to 20.41] ^*^ | .03 [.01 to .09] ^*^ |
| Moderate Movers (77.6) | .02 [.02 to .03] ^*^ | ref. | .01 [.01 to .02] ^*^ |
| Minimal Movers (12.6) | .12 [.04 to .34] ^*^ | 17.62 [12.57 to 24.69] ^*^ | ref. |

*^*^ Odds adjusted for accelerometer wear time, sex, BMI, parental education and baseline depressive symptoms*

## Predictors of Profile Transitions: Effects of Each Predictor Tested Simultaneously

|  |  | Wave 2 | | |
| --- | --- | --- | --- | --- |
| Sex | Wave 1 | Maximal Movers | Moderate Movers | Minimal Movers |
|  | Maximal Movers | ref. | 2.126(1.692,2.671)^*^ | 3.530(2.611,4.772)^*^ |
|  | Moderate Movers | 0.470(0.374,0.591)^*^ | ref. | 1.660(1.338,2.060)^*^ |
|  | Minimal Movers | 0.283(0.210,0.383)^*^ | 0.602(0.485,0.747)^*^ | ref. |
|  |  | Wave 3 | | |
|  | Wave 2 | Maximal Movers | Moderate Movers | Minimal Movers |
|  | Maximal Movers | ref. | 1.345(0.981,1.844) | 1.784(1.171,2.718)^*^ |
|  | Moderate Movers | 0.744(0.542,1.020) | ref. | 1.327(0.993,1.772) |
|  | Minimal Movers | 0.560(0.368,0.854)^*^ | 0.754(0.564,1.007) | ref. |
|  |  | Wave 2 | | |
| BMI | Wave 1 | Maximal Movers | Moderate Movers | Minimal Movers |
|  | Maximal Movers | ref. | 1.042(1.005,1.080)^*^ | 1.060(1.013,1.109)^*^ |
|  | Moderate Movers | 0.960(0.926,0.995)^*^ | ref. | 1.017(0.987,1.048)^*^ |
|  | Minimal Movers | 0.944(0.902,0.987)^*^ | 0.983(0.955,1.013) | ref. |
|  |  | Wave 3 | | |
|  | Wave 2 | Maximal Movers | Moderate Movers | Minimal Movers |
|  | Maximal Movers | ref. | 0.987(0.945,1.031) | 0.953(0.896,1.014) |
|  | Moderate Movers | 1.013(0.970,1.058) | ref. | 0.965(0.922,1.010) |
|  | Minimal Movers | 1.049(0.986,1.117) | 1.036(0.990,1.084) | ref. |
| Parental |  | Wave 2 | | |
| Education | Wave 1 | Maximal Movers | Moderate Movers | Minimal Movers |
|  | Maximal Movers | ref. | 1.142(1.038,1.255)^*^ | 1.350(1.184,1.540)^*^ |
|  | Moderate Movers | 0.876(0.797,0.963)^*^ | ref. | 1.183(1.071,1.305)^*^ |
|  | Minimal Movers | 0.741(0.650,0.845)^*^ | 0.846(0.766,0.934)^*^ | ref. |
|  |  | Wave 3 | | |
|  | Wave 2 | Maximal Movers | Moderate Movers | Minimal Movers |
|  | Maximal Movers | ref. | 1.021(0.893,1.167) | 1.089(0.912,1.300) |
|  | Moderate Movers | 0.980(0.857,1.120) | ref. | 1.067(0.945,1.204) |
|  | Minimal Movers | 0.919(0.769,1.097) | 0.938(0.831,1.058) | ref. |
| Baseline |  | Wave 2 | | |
| SMFQ | Wave 1 | Maximal Movers | Moderate Movers | Minimal Movers |
|  | Maximal Movers | ref. | 1.001(0.971,1.032) | 1.006(0.968,1.045) |
|  | Moderate Movers | 0.999(0.969,1.030) | ref. | 1.005(0.980,1.031) |
|  | Minimal Movers | 0.994(0.957,1.033) | 0.995(0.970,1.021) | ref. |
|  |  | Wave 3 | | |
|  | Wave 2 | Maximal Movers | Moderate Movers | Minimal Movers |
|  | Maximal Movers | ref. | 1.003(0.960,1.047) | 1.022(0.969,1.079) |
|  | Moderate Movers | 0.997(0.955,1.041) | ref. | 1.019(0.986,1.054) |
|  | Minimal Movers | 0.978(0.927,1.032) | 0.981(0.949,1.014) | ref. |

^*^*significantly predicted transition probability*

## Predictors of Profile Transitions: Effects of Each Predictor Tested Independently in Separate Models

|  |  | Wave 2 | | |
| --- | --- | --- | --- | --- |
| Sex | Wave 1 | Maximal Movers | Moderate Movers | Minimal Movers |
|  | Maximal Movers | ref. | 2.163(1.724,2.715)^*^ | 3.621(2.682,4.888)^*^ |
|  | Moderate Movers | 0.462(0.368,0.580)^*^ | ref. | 1.674(1.350,2.076)^*^ |
|  | Minimal Movers | 0.276(0.205,0.373)^*^ | 0.597(0.482,0.741)^*^ | ref. |
|  |  | Wave 3 | | |
|  | Wave 2 | Maximal Movers | Moderate Movers | Minimal Movers |
|  | Maximal Movers | ref. | 1.343(0.981,1.838) | 1.774(1.165,2.702)^*^ |
|  | Moderate Movers | 0.745(0.544,1.020) | ref. | 1.322(0.988,1.767) |
|  | Minimal Movers | 0.564(0.370,0.858)^*^ | 0.757(0.566,1.012) | ref. |
|  |  | Wave 2 | | |
| BMI | Wave 1 | Maximal Movers | Moderate Movers | Minimal Movers |
|  | Maximal Movers | ref. | 1.049(1.012,1.087)^*^ | 1.065(1.018,1.113)^*^ |
|  | Moderate Movers | 0.953(0.920,0.989)^*^ | ref. | 1.015(0.986,1.045) |
|  | Minimal Movers | 0.939(0.898,0.982)^*^ | 0.985(0.957,1.014) | ref. |
|  |  | Wave 3 | | |
|  | Wave 2 | Maximal Movers | Moderate Movers | Minimal Movers |
|  | Maximal Movers | ref. | 0.989(0.947,1.033) | 0.957(0.899,1.017) |
|  | Moderate Movers | 1.011(0.968,1.056) | ref. | 0.967(0.925,1.012) |
|  | Minimal Movers | 1.045(0.983,1.112) | 1.034(0.989,1.081) | ref. |
| Parental |  | Wave 2 | | |
| Education | Wave 1 | Maximal Movers | Moderate Movers | Minimal Movers |
|  | Maximal Movers | ref. | 1.131(1.028,1.244)^*^ | 1.328(1.165,1.513)^*^ |
|  | Moderate Movers | 0.884(0.804,0.973)^*^ | ref. | 1.174(1.065,1.295)^*^ |
|  | Minimal Movers | 0.753(0.661,0.858)^*^ | 0.852(0.772,0.939)^*^ | ref. |
|  |  | Wave 3 | | |
|  | Wave 2 | Maximal Movers | Moderate Movers | Minimal Movers |
|  | Maximal Movers | ref. | 1.027(0.898,1.176) | 1.111(0.930,1.327) |
|  | Moderate Movers | 0.973(0.851,1.113) | ref. | 1.081(0.959,1.219) |
|  | Minimal Movers | 0.900(0.754,1.075) | 0.925(0.820,1.043) | ref. |
| Baseline |  | Wave 2 | | |
| SMFQ | Wave 1 | Maximal Movers | Moderate Movers | Minimal Movers |
|  | Maximal Movers | ref. | 1.011(0.981,1.043) | 1.026(0.988,1.065) |
|  | Moderate Movers | 0.989(0.959,1.019) | ref. | 1.014(0.989,1.040) |
|  | Minimal Movers | 0.975(0.939,1.012) | 0.986(0.962,1.011) | ref. |
|  |  | Wave 3 | | |
|  | Wave 2 | Maximal Movers | Moderate Movers | Minimal Movers |
|  | Maximal Movers | ref. | 1.006(0.964,1.051) | 1.028(0.975,1.085) |
|  | Moderate Movers | 0.994(0.952,1.037) | ref. | 1.022(0.988,1.056) |
|  | Minimal Movers | 0.973(0.922,1.026) | 0.979(0.947,1.012) | ref. |

^*^*significantly predicted transition probability*

## Differences in Depressive Symptoms at Age 18 and 22 according to Profile Membership at T3

|  |  |  | SMFQ | Wald Test^a^ |  |  | SMFQ | Wald Test^a^ |  |  |
| --- | --- | --- | --- | --- | --- | --- | --- | --- | --- | --- |
|  | T3 Profile | N (%) | at 18 (*SE*) | (*SE*) | *p* | *d* | at 22 (*SE*) | (SE) | *p* | *d* |
| Unadjusted | Minimal | 259 (5.2) | 5.92 (.32) | ref. |  |  | 4.66 (.31) | ref. |  |  |
|  | Moderate | 4512 (90.1) | 5.21 (.07) | -.72 (.33)^*^ | .03 | .16 | 2.57 (.06) | -1.091 (.32)^*^ | <.01 | .26 |
|  | Maximal | 193 (3.9) | 4.87 (.30) | -1.05 (.44)^*^ | .02 | .24 | 3.36 (.33) | -1.296 (.46)^*^ | <.01 | .31 |
|  |  |  |  |  |  |  |  |  |  |  |
|  | Moderate |  |  | ref. |  |  |  | ref. |  |  |
|  | Maximal |  |  | -.33 (.30) | .27 | .08 |  | -.205 (.34) | .55 | .05 |
| Fully | Minimal | 259 (5.2) | 5.94 (.32) | ref. |  |  | 4.70 (.32) | ref. |  |  |
| Adjusted ^b^ | Moderate | 4512 (90.1) | 5.20 (.07) | -.74 (.33)^*^ | .03 | .17 | 3.56 (.06) | -1.14 (.33)^*^ | <.01 | .27 |
|  | Maximal | 193 (3.9) | 4.87 (.30) | -1.07 (.44)^*^ | .02 | .25 | 3.36 (.34) | -1.34 (.47)^*^ | <.01 | .32 |
|  |  |  |  |  |  |  |  |  |  | .05 |
|  | Moderate |  |  | ref. |  |  |  | ref. |  |  |
|  | Maximal |  |  | -.34 (.31) | .28 | .08 |  | -.20 (.34) | .55 |  |

^*^*significant difference*

*^a^ Wald tests subtracted reference group mean/intercept from comparison group mean/intercept hence, a negative value indicates comparison group had fewer symptoms, a positive value indicates comparison group had greater symptoms*

*^b^ adjusted for sex, BMI, parental education, baseline depressive symptoms*

## Differences in Depressive Symptoms at Age 18 and 22 according to Latent Transition Probabilities

|  | Transition |  | SMFQ | Wald Test Statistics ^a^ | | | SMFQ | Wald Test Statistics ^a^ | | | |
| --- | --- | --- | --- | --- | --- | --- | --- | --- | --- | --- | --- |
|  | Pattern | *n* (%) | at 18 (*SE*) | (*SE*) | *p* | *d* | at 22 (*SE*) | (*SE*) | *p* | *d* |  |
| Unadjusted | 1→1→2 | 123 (2.5) | 4.45 (.39) | -.67 (.40) | .10 | .16 | 2.85 (.34) | -.68 (.36) | .06 | .16 |  |
|  | 1→2→2 | 562 (11.3) | 4.55 (.18) | -.57 (.21)^*^ | <.01 | .13 | 2.67 (.16) | -.85 (.19)^*^ | <.001 | .20 |  |
|  | 2→1→2 | 262 (5.3) | 4.90 (.29) | -.23 (.31) | .46 | .05 | 3.19 (.23) | .33 (.25) | .19 | .08 |  |
|  | 2→2→2 | 2,346 (47.2) | 5.12 (.10) | ref. |  |  | 3.52 (.10) | ref. |  |  |  |
|  | 2→3→2 | 244 (4.9) | 6.24 (.38) | 1.12 (.41)^*^ | <.01 | .26 | 4.47 (.43) | .95 (.45)^*^ | .04 | .23 |  |
|  | 3→2→2 | 761 (15.3) | 5.58 (.19) | .46 (.23)^*^ | .04 | .11 | 3.93 (.19) | .41 (.23) | .07 | .10 |  |
|  | 3→3→2 | 210 (4.2) | 5.92 (.36) | .80 (.37)^*^ | .03 | .19 | 4.99 (.45) | 1.47 (.46)^*^ | <.01 | .35 |  |
| Fully | 1→1→2 | 121 (2.4) | 1.52 (.51) | -.32 (.33) | .34 | .08 | -.15 (.51) | -.34 (.33) | .30 | .09 |  |
| Adjusted ^b^ | 1→2→2 | 566 (11.4) | 1.58 (.45) | .26 (.20) | .20 | .06 | -.39 (.44) | -.58 (.18)^*^ | <.01 | .14 |  |
|  | 2→1→2 | 269 (5.4) | 1.97 (.51) | .13 (.29) | .67 | .03 | .13 (.47) | -.06 (.25) | .81 | .02 |  |
|  | 2→2→2 | 2,338 (47.1) | 1.84 (.45) | ref. |  |  | .19 (.44) | ref. |  |  |  |
|  | 2→3→2 | 244 (4.9) | 2.61 (.58) | .77 (.38)^*^ | .04 | .19 | .81 (.60) | .62 (.42) | .14 | .16 |  |
|  | 3→2→2 | 766 (15.4) | 2.04 (.48) | .20 (.22) | .35 | .05 | .32 (.48) | .13 (.22) | .56 | .03 |  |
|  | 3→3→2 | 204 (4.1) | 2.48 (.58) | .64 (.36) | .08 | .16 | 1.50 (.65) | 1.31 (.47)^*^ | <.01 | .33 |  |

^*^*significant difference*

*^a^ Wald tests subtracted reference group mean/intercept from comparison group mean/intercept hence, a negative value indicates comparison group had fewer symptoms, a positive value indicates comparison group had greater symptoms*

*^b^ adjusted for sex, BMI, parental education, baseline depressive symptoms*

# Sensitivity Analysis: Complete Case (Attrition) Analysis

| Classes | LL | AIC | BIC | ssaBIC | LMRa | Entropy | Class Proportions Based on Estimated Posterior Probabilities (%) |
| --- | --- | --- | --- | --- | --- | --- | --- |
| Age 12 |  |  |  |  |  |  |  |
| 1 | -12896 | 25805 | 25833 | 25814 | - | - | 100 |
| 2 | -12687 | 25394 | 25441 | 25409 | .000 | .653 | 58, 42 |
| 3 | -12594 | 25217 | 25284 | 25240 | .000 | .730 | 56, 29, 15 |
| 4 | -12543 | 25123 | 25209 | 25152 | .142 | .759 | 54, 30, 10, 6 |
| 5 | -12514 | 25073 | 25178 | 25108 | .154 | .759 | 44, 38, 8, 6, 4 |
| 6 | -12483 | 25018 | 25141 | 25059 | .069 | .773 | 39, 38, 7, 6, 5, 5 |
| 7 | -12461 | 24983 | 25126 | 25030 | .000 | .791 | 38, 37, 8, 7, 5, 5, 1 |
| 8 | -12449 | 24966 | 25128 | 25020 | .727 | .756 | 34, 27, 18, 8, 7, 4, 1, 1 |
| 9 | -12438 | 24953 | 25134 | 25013 | .130 | .778 | 36, 24, 16, 6, 5, 5, 3, 3, 1 |
| 10 | -12427 | 24939 | 25139 | 25005 | .204 | .776 | 37, 23, 16, 7, 6, 5, 3, 1, 1, 1 |
| Age 14 |  |  |  |  |  |  |  |
| 1 | -13027 | 26066 | 26095 | 26076 | - | - | 100 |
| 2 | -12805 | 25630 | 25678 | 25646 | .000 | .732 | 69, 31 |
| 3 | -12742 | 25512 | 25579 | 25534 | .000 | .683 | 55, 26, 18 |
| 4 | -12701 | 25438 | 25523 | 25466 | .044 | .724 | 55, 25, 14, 7 |
| 5 | -12670 | 25384 | 25489 | 25419 | .161 | .757 | 52, 26, 12, 7, 3 |
| 6 | -12653 | 25359 | 25483 | 25401 | .431 | .735 | 38, 26, 16, 12, 7, 1 |
| 7 | -12636 | 25333 | 25476 | 25380 | .140 | .759 | 38, 27, 16, 8, 8, 4, 1 |
| 8 | -12626 | 25321 | 25483 | 25375 | .446 | .720 | 36, 19, 16, 13, 8, 5, 4, 1 |
| 9 | -12611 | 25299 | 25480 | 25359 | .125 | .744 | 36, 17, 17, 14, 8, 4, 4, 3, 1, <1 |
| 10 | -12601 | 25286 | 25485 | 25352 | .105 | .754 | 36, 16, 15, 15, 7, 4, 4, 4, 1, <1 |
| Age 16 |  |  |  |  |  |  |  |
| 1 | -13041 | 26095 | 26123 | 26104 | - | - | 100 |
| 2 | -12895 | 25810 | 25857 | 25826 | .000 | .716 | 80, 20 |
| 3 | -12824 | 25676 | 25742 | 25698 | .005 | .826 | 78, 13, 8 |
| 4 | -12783 | 25603 | 25688 | 25631 | .000 | .714 | 54, 30, 12, 5 |
| 5 | -12758 | 25561 | 25666 | 25596 | .113 | .736 | 49, 30, 9, 8, 4 |
| 6 | -12732 | 25517 | 25640 | 25558 | .140 | .756 | 43, 28, 12, 10, 4, 3 |
| 7 | -12721 | 25503 | 25646 | 25550 | .417 | .756 | 44, 26, 11, 10, 4, 3, 2 |
| 8 | -12711 | 25491 | 25653 | 25545 | .183 | .768 | 43, 26, 12, 10, 4, 3, 2, 1 |
| 9 | -12701 | 25478 | 25659 | 25538 | .048 | .776 | 42, 25, 12, 10, 3, 3, 2, 2, 1 |
| 10 | -12693 | 25470 | 25670 | 25536 | .240 | .784 | 42, 24, 11, 10, 3, 3, 3, 2, 2, 1 |

## Model Fit Indices for Cross-Sectional Latent Profiles of Adolescent Movement Behaviour Controlling for Wear Time (Complete Case)

## Elbow Plots Illustrating Model Fit of Cross-Sectional Latent Profile Models with k+1 Solutions (Complete Case)

**
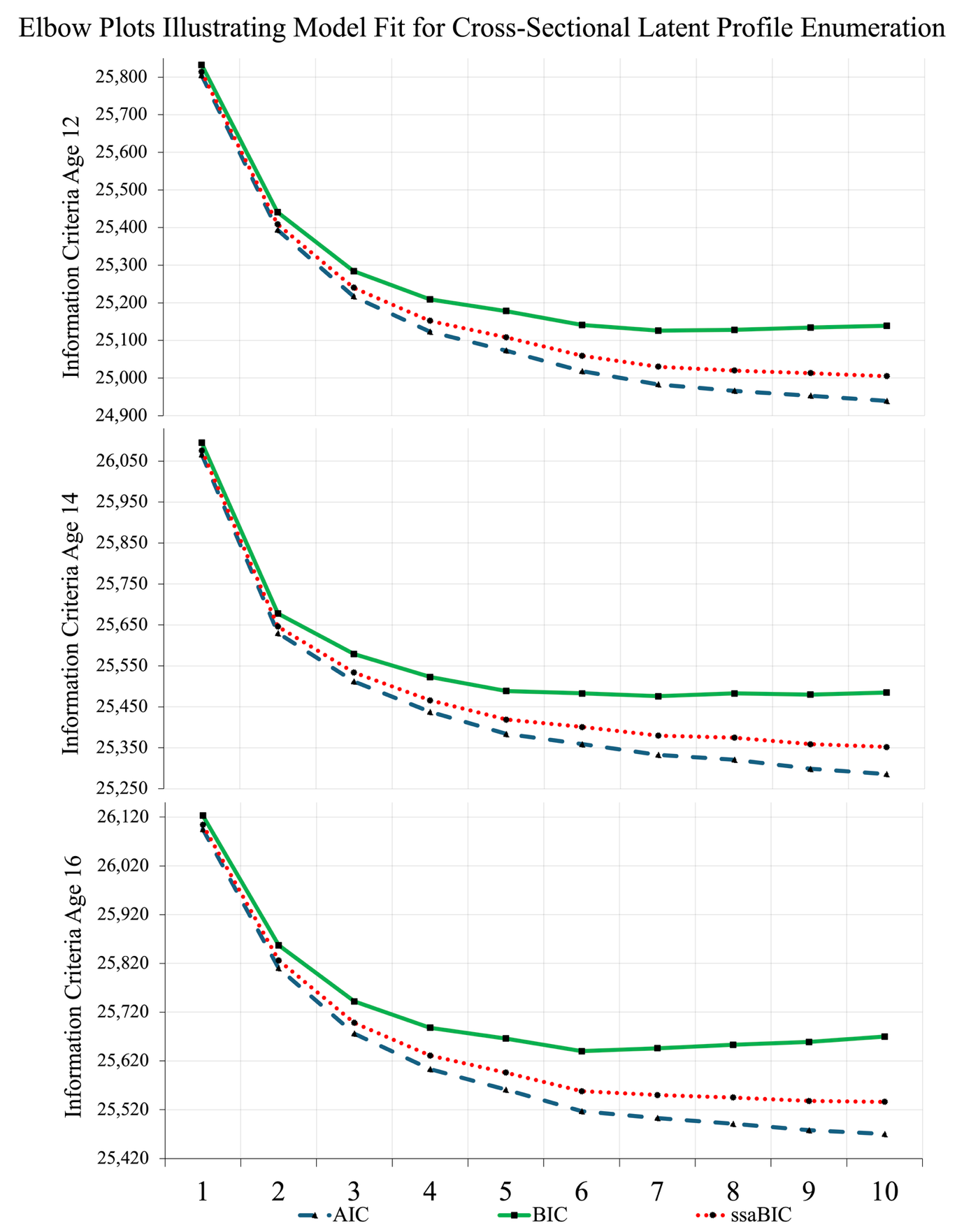
**

## Invariance Testing: Satorra-Bentler Scaled (Mean-Adjusted) Chi-Square Difference Test

1. Estimate the nested and comparison models in Mplus using MLR. The output provides loglikelihood values L^0^ and L^1^ and scaling correction factors c^0^ and c^1^ for the H^0^ and H^1^ models:

|  | Regular LTA models | RI-LTA models |
| --- | --- | --- |
| L^0^ | -38189.842 | -37897.006 |
| c^0^ | 1.2208 | 1.2758 |
| p^0^ | 38 | 41 |
| L^1^ | -37963.713 | -37635.588 |
| c^1^ | 1.2424 | 1.4444 |
| p^1^ | 56 | 59 |

1. Formula used to compute the difference test scaling correction where p^0^ is the number of parameters in the nested model and p^1^ is the number of parameters in the comparison model.

cd = (p^0^ * c^0^ - p^1^*c^1^)/(p^0^ - p^1^)

TRd = -2*(L^0^ - L^1^)/cd

df = p1 – p0

1. Compare result against a chi square distribution table for the number of df where *p* < .05

Table found at: <https://www.medcalc.org/manual/chi-square-table.php>

### Comparison of Regular LTA models:

cd = (38 * 1.2208 – 56 * 1.2424)/(38 - 56) = 1.228

TRd = -2*(-38189.842 + 37963.713)/1.228 = 368.14

df = 56 – 38 = 18

The test result of 368.14 exceeds the critical value on a chi-square distribution for *df*=18 (22.869) meaning the test result is highly significant, suggesting the non-invariant (comparison) model yields a significantly better fit to the data than the invariant (nested) model. The null hypothesis of invariance was rejected, and parameters were allowed to vary over time.

### Comparison of RI-LTA models:

cd = (41 * 1.2758 – 59 * 1.444)/(41 - 59) = 1.827

TRd = -2*(-37897.006 + 37635.588)/1.827 = 286.06

df = 59 – 41 = 18

The test result of 286.06 exceeds the critical value on a chi-square distribution for *df*=18 (22.869) meaning the test result is highly significant, suggesting that the non-invariant (comparison) model yields a significantly better fit to the data than the invariant (nested) model. The null hypothesis of invariance was rejected, and parameters were allowed to vary over time.

## Fit of Invariant and Non-Invariant Regular LTA and RI-LTA Models (Complete Case)

| Model | BIC | Satorra-Bentler  (*p*) | Entropy | Estimated Class Proportions Based on Posterior Probabilities (%) |
| --- | --- | --- | --- | --- |
| Regular LTA | 76636.360 | - | Overall = .772 |  |
| (invariant) |  |  | T1 = .768 | T1 = 51, 46, 3 |
|  |  |  | T2 = .751 | T2 = 52, 32, 16 |
|  |  |  | T3 = .756 | T3 = 65, 30, 4 |
| RI-LTA | 76070.952 | - | Overall = .763 |  |
| (invariant) |  |  | T1 = .710 | T1 = 57, 38, 5 |
|  |  |  | T2 = .686 | T2 = 49, 45, 6 |
|  |  |  | T3 = .799 | T3 = 84, 14, 2 |
| Regular LTA | 76305.684 | < .05 | Overall = .754 |  |
| (non-invariant) |  |  | T1 = .748 | T1 = 55, 29, 16 |
|  |  |  | T2 = .724 | T2 = 48, 34, 18 |
|  |  |  | T3 = .755 | T3 = 48, 46, 6 |
| RI-LTA | 75669.697 | < .05 | Overall = .730 |  |
| (non-invariant) |  |  | T1 = .684 | T1 = 55, 30, 15 |
|  |  |  | T2 = .806 | T2 = 66, 28, 6 |
|  |  |  | T3 = .662 | T3 = 43, 36, 21 |

## Minimally Adjusted 3x3 Non-Invariant RI-LTA Model, with Movement Behaviours Expressed as the Proportion of Daily Accelerometer Wear Time (Complete Case; %)


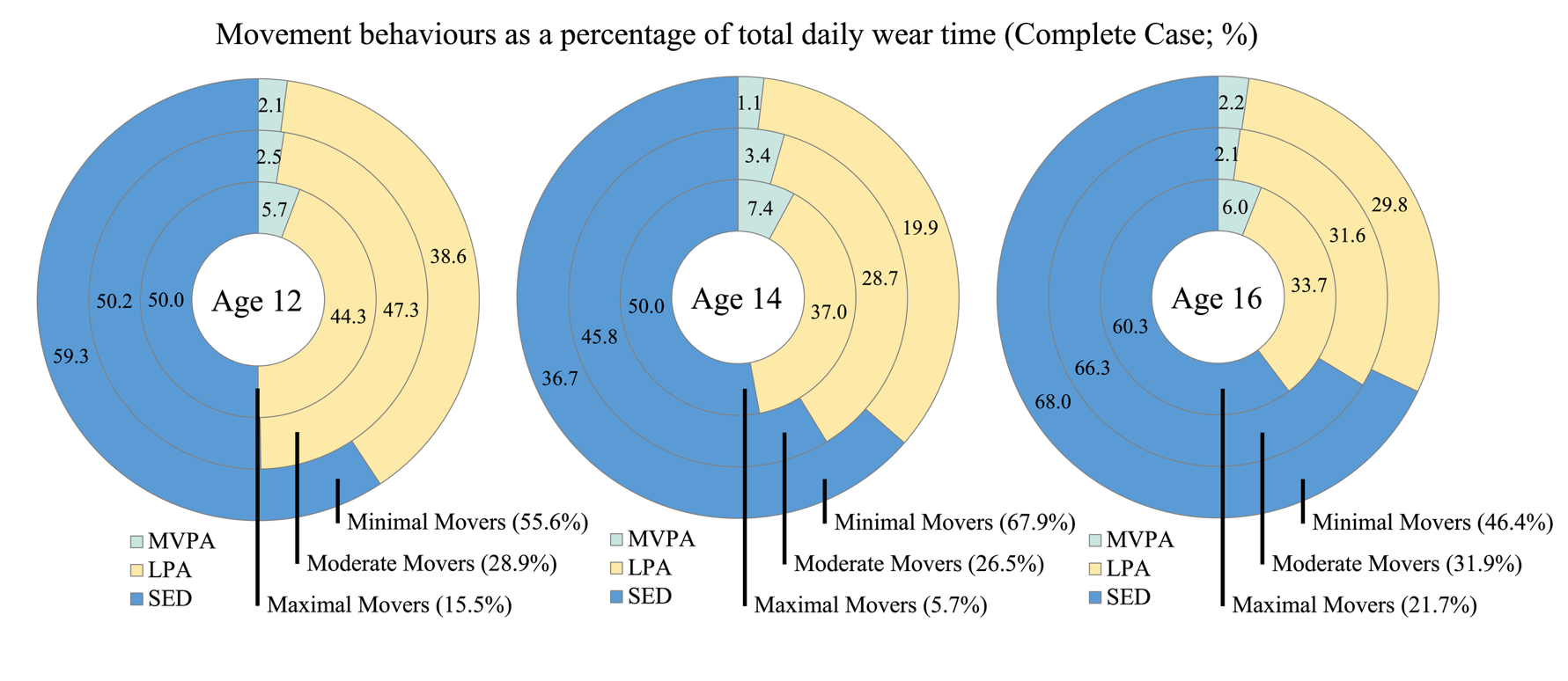

Supplement: Supplementary file 1 — Supplementary Material 1 [file 41598_2025_4466_MOESM1_ESM.docx]
